# Linkage mapping evidence for a syntenic QTL associated with flowering time in perennial C_4_ rhizomatous grasses *Miscanthus* and switchgrass

**DOI:** 10.1111/gcbb.12755

**Published:** 2020-10-28

**Authors:** Elaine Jensen, Reza Shafiei, Xue‐Feng Ma, Desalegn D. Serba, Daniel P. Smith, Gancho T. Slavov, Paul Robson, Kerrie Farrar, Sian Thomas Jones, Timothy Swaller, Richard Flavell, John Clifton‐Brown, Malay C. Saha, Iain Donnison

**Affiliations:** ^1^ Institute of Biological, Environmental and Rural Sciences Aberystwyth University Aberystwyth UK; ^2^ University of Dundee at JHI Dundee UK; ^3^ Ceres, Inc. Thousand Oaks CA USA; ^4^ Noble Research Institute, LLC. Ardmore OK USA; ^5^ Agricultural Research Center‐Hays Kansas State University Hays KS USA; ^6^ Scion Rotorua New Zealand; ^7^ Genomics Institute of the Novartis Research Foundation San Diego CA USA; ^8^ International Wheat Yield Partnership Texas A&M University College Station TX USA

**Keywords:** bioenergy, floral transition conservation, heading date quantitative trait loci, *Panicum virgatum*, perennial biomass crop breeding

## Abstract

Flowering in perennial species is directed via complex signalling pathways that adjust to developmental regulations and environmental cues. Synchronized flowering in certain environments is a prerequisite to commercial seed production, and so the elucidation of the genetic architecture of flowering time in *Miscanthus* and switchgrass could aid breeding in these underdeveloped species. In this context, we assessed a mapping population in *Miscanthus* and two ecologically diverse switchgrass mapping populations over 3 years from planting. Multiple flowering time quantitative trait loci (QTL) were identified in both species. Remarkably, the most significant *Miscanthus* and switchgrass QTL proved to be syntenic, located on linkage groups 4 and 2, with logarithm of odds scores of 17.05 and 21.8 respectively. These QTL regions contained three flowering time transcription factors: *Squamosa Promoter‐binding protein‐Like*, MADS‐box *SEPELLATA2* and gibberellin‐responsive bHLH137. The former is emerging as a key component of the age‐related flowering time pathway.

## Graphical abstract

Genetic factors control flowering in the bioenergy species *Miscanthus* and switchgrass are poorly understood, yet commercial seed production to upscale sustainable bioenergy requires flowering synchronization. Using populations (of both species) with diverse flowering times, we identified a region that was strikingly conserved, not only between *Miscanthus* and switchgrass, but also with Sorghum, another bioenergy grass. The region contained DNA sequences encoding genes known to be involved in flowering time control in other species, and appears highly important for flowering time regulation. This provides a potential tool to synchronize flowering in these crops to speed up the breeding of new varieties.

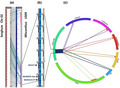

## 1 INTRODUCTION

C_4_ biomass grasses such as sugarcane (*Saccharum officinarum*), sorghum (*Sorghum bicolor*), *Miscanthus* (*Miscanthus* spp.) and switchgrass (*Panicum virgatum*) are some of the most photosynthetically efficient species in the plant kingdom. Feedstock from these crops are suitable for conversion into energy and bio‐products, a promising approach to curbing greenhouse gas (GHG) emissions and providing sustainable alternatives to petrochemical products (Clark et al., 2009; Hastings et al., 2008; McCalmont et al., 2017; Robson et al., 2019).

*Miscanthus* and switchgrass are widely cultivated perennial bioenergy crops in Europe and the United States, respectively, and capable of producing moderate to high biomass yields with low inputs, even on marginal or contaminated land (Clifton‐Brown et al., 2001; Hope & McElroy, 1990; McLaughlin et al., 1999; Moser & Vogel., 1995; Purdy et al., 2013; Rusinowski et al., 2019).

*Miscanthus* demonstrates stand and harvest longevity exceeding 15 years (Clifton‐Brown et al., 2019) and, once established, expresses considerable drought (Scordia et al., 2020) and flood (Kam et al., 2020) tolerance with low to zero requirements for herbicide treatments and fertilization (Cosentino et al., 2007). The commercially dominant triploid *Miscanthus* x *giganteus* (*Mxg*) is a natural interspecific hybrid between diploid *Miscanthus sinensis* (2n = 2x = 38) and allotetraploid *Miscanthus* *sacchariflorus* (2n = 4x = 76; Greef & Deuter, 1993; Linde‐Laursen, 1993; Rayburn et al., 2009). The triploid nature of *Mxg* means that propagation is vegetative. The monoclonal nature of this crop therefore poses risks for disease outbreaks or yield losses due to abiotic stresses (Lewandowski et al., 2016). It is therefore essential that we broaden the genetic base of commercially deployed *Miscanthus* to address vulnerabilities and improve yield quality and quantity. Furthermore, recent Net Zero policies (Committee on Climate Change, 2020) recommend the scaling up of planting rates of energy crops like *Miscanthus* to deliver CO_2_‐equivalent emissions savings. Both of these objectives can be achieved through the development of seed‐based hybrid varieties (Clifton‐Brown et al., 2017, 2019) using diverse *Miscanthus* genotypes.

Switchgrass comprises two major ecotypes believed to have diverged approximately 0.8–1.0 Mya (Morris et al., 2011; Young et al., 2011; Zhang et al., 2011), which are adapted to either upland or lowland environments. Differences in ecotype morphology and habitat preferences are believed to result from the combined effects of broad species adaptation and microevolutionary processes. The upland ecotype (predominantly octoploid 2n = 8x = 72, with some tetraploid) is typical of the mid to Northern United States. It has short fine stems and matures early. The vigorous lowland ecotype (tetraploid 2n = 4x = 36) largely grows in the warmer and moist southern United States and has thick and tall stems, which mature later than the upland ecotype (Vogel et al., 1985).

The extensive phenotypic and genotypic diversity among *Miscanthus* and switchgrass genera provides broad adaptability to a wide range of soil and climatic conditions. This same variety also provides diverse germplasm for genetic studies of adaptive and agronomically important traits, including flowering time (Bouton, 2007; Casler, 2012; Clark et al., 2016, 2019; Jensen et al., 2013; Robson et al., 2013; Slavov et al., 2014)—a key trait for yield intensification (Casler, 2019; Jensen et al., 2011, 2013; Taylor et al., 2018). However, flowering time is a complex trait and the broad phenotypic variation exhibited in *Miscanthus* and switchgrass (Jensen et al., 2011; Schwartz & Amasino, 2013) is probably underpinned by multiple genes.

Flowering time genetic control differs between annuals and perennials (Amasino, 2009; Friedman & Rubin, 2015; Khan et al., 2014; Kiefer et al., 2017; Nuñez & Yamada, 2017). The annual plant model Arabidopsis operates five known flowering pathways that respond to a range of developmental regulators and seasonal cues (Andres & Coupland, 2012; Bratzel & Turck, 2015; Hyun et al., 2017; Wils & Kaufmann, 2017). The exogenous (seasonal) cues regulate pathways associated with photoperiod and vernalization, while endogenous signals regulate the gibberellin, autonomous and ageing pathways. The latter prevents flowering during the juvenile phase but can also trigger the transition to flowering if other inducing conditions fail (Teotia & Tang, 2015; Wang et al., 2009). All these pathways integrate flowering time by converging on a common set of downstream genes, such as FLOWERING LOCUS C, FLOWERING LOCUS T and the transcription factor LEAFY. In perennial grasses like *Miscanthus* and switchgrass, the understanding of flowering time control is in its infancy; genetic analyses are relatively recent and model systems are less well developed compared to Arabidopsis.

However, a number of genetic maps have now been constructed in *Miscanthus* (Atienza et al., 2002; Kim et al., 2012; Liu et al., 2016; Ma et al., 2012) and switchgrass (Liu et al., 2012; Okada et al., 2010; Serba et al., 2013) permitting the alignment of their linkage groups (LGs) to other species, and enabling the development of breeding tools such as quantitative trait loci (QTL) and, potentially, marker assisted selection (MAS). Due to the relatively more recent interest in *Miscanthus* and switchgrass, and the added difficulties of genotyping and genetic mapping outcrossing (self‐incompatible) species with large and highly heterozygous genomes, the development of QTL and MAS is behind that of many other species. Nevertheless, flowering time QTL have now been identified in both species (Ali et al., 2019; Ge et al., 2019; Gifford et al., 2015; Tornqvist et al., 2018), and the process of identifying genes regulating flowering time control can begin.

We utilized the *M. sinensis* genetic map developed by Ma et al. (2012), as well as two maps from switchgrass: one published by Serba et al. (2013), and a second from a Noble‐Ceres mapping family, which is proprietary to Ceres, Inc., and segregates for many agronomic traits affecting yield. We combined phenotypic data from replicated field trials to perform parallel QTL scans for flowering time in these two biomass crops, thereby also enabling more general analyses of synteny and colinearity between the Andropogoneae and Paniceae tribes.

## 2 MATERIALS AND METHODS

### 2.1 Experimental populations

Table S1 shows the three full‐sib mapping populations that were studied. The *M. sinensis* Mx2 population is an outcrossing family from two heterozygous parental lines that expressed phenotypic variation in flowering time, as described previously (Ma et al., 2012). This map was constructed using genotyping‐by‐sequencing (GBS)‐derived SNP markers (Ma et al., 2012). The AP13 × VS16 is a full‐sib pseudo‐testcross and was derived from a cross between a lowland Alamo genotype: AP13 as a female, and an upland Summer genotype, VS16 as a male parent (Missaoui et al., 2005; Serba et al., 2013). The SG2 × SG1 is also a full‐sib pseudo‐testcross mapping population. The two parental genotypes were selected, based on their distinct morphologies, from a natural lowland population collected from Tennessee, United States. The SG1 plant had short, yellowish‐green leaves and a sprawling growth habit, while the SG2 plant was tall, with many stems and blue‐green leaves. The map was constructed using markers in common with the AP13 × VS16 map.

### 2.2 Field experiments

The field trial for the *Miscanthus* mapping family was established near Aberystwyth (52.43°N, 3.99°W), on the west coast of Wales, United Kingdom. In May 2007, three clonal replicates generated from each of the 216 genotypes were planted following a randomized complete block design with three replications. Planting spaces were 1.5 m between rows and columns. Positions on the perimeter of the trial area and between blocks were planted with *Mx*g. An automatic weather station at the trial site provided the daily climate data, that is, rainfall, temperature, radiation and humidity. Heat accumulation, in degree days, was calculated on a daily time step above a threshold temperature of 10°C, using equations described previously (McVicker, 1946). Pre‐emergence glyphosate (2 L/ha) and postemergence Stomp^®^ Aqua (1.5 L/ha) herbicides were used for weed control until the site was well established. NPK fertilizer (14:14:21) was applied at a rate of 11.3 g per plant in the second year.

The field experiment for the AP13 × VS16 and SG2 × SG1 switchgrass mapping populations was conducted at two locations in southern Oklahoma, United States. The two locations were Noble Research Park at Ardmore, OK (34.1120°N, 97.5376°W, soil type: Alfisolsa Wilson silt loam soil) and the Noble Red River Farm near Burneyville, OK (33.9079°N, 97.2889°W, soil type: Mollisolsa Minc fine sandy loam). The field experimental designs of the two populations were the same. A total of 251 full‐sib progeny, duplicates of the parental genotypes, and an Alamo genotype were evaluated in an R‐256 honeycomb design (Fasoula & Fasoula, 2000) with four replications. The field experiments at Burneyville and Ardmore were transplanted on April 22, 2008 and August 27, 2008 respectively. Plant spacing was maintained at 1.5 m between plants with a row spacing of 1.3 m and even rows staggered at 0.75 m. The seedbed preparation, establishment, fertilizer application, supplemental irrigation, weed control and interplant cultivation was as described by Serba et al. (2015).

### 2.3 Phenotypic data

Flowering times in *Miscanthus* were recorded in 2009, 2010 and 2011. Flowering development was divided into four flowering stages (FS1–4) as described previously (Jensen et al., 2011). Briefly, FS1 was the day of year (DOY) when the first flag leaves emerged; FS2 was the DOY when 1 cm or more of the panicle was showing on at least one stem; FS3 was the DOY when approximately 50% of stems contributing to canopy height had exerted more than 1 cm of panicle; and FS4 was the DOY when more than 80% of the stems contributing to canopy height had exerted greater than 1 cm of the panicle. These stages were denoted as ‘FS*n*DOY’ where *n* is the number of the flowering stage. In addition to the above, in 2010 and 2011 the DOY when anthesis commenced (AN) was also recorded. Data were collected from the day the first flag leaf became visible until no further flowering development was observed at the end of the season. Observations were made two to three times a week during the flowering period.

Heading in switchgrass was recorded as a proxy trait for flowering (in general, flowering is 2–4 days after heading), for ease of recording, and was collected at Ardmore and Burneyville in 2009 and 2010. Heading date was recorded when approximately 50% of the tillers in a plant showed inflorescence emergence, meaning that flowering had already started in some of the plants. Regrowth date was scored for each plant when new shoots came out of the crown in the spring. The number of days elapsed between the regrowth and heading dates was considered the length of the vegetative growth period.

### 2.4 Linkage maps

The genetic map construction for the *M. sinensis* mapping population was described previously (Ma et al., 2012). Briefly, the composite map comprised 3,745 GBS‐derived SNP markers spanning 2,396 cM on 19 LGs with an average resolution of 0.64 cM. For switchgrass, we used the Noble‐Ceres AP13 × VS16 map, which was constructed independently by Ceres, Inc., using a different set of markers to those published previously (Serba et al., 2015). Both the AP13 × VS16 and SG2 × SG1 maps identified all 18 LGs expected for the tetraploid switchgrass. The homologous linkage relation between the two maps was also established based on common markers and common alignment to a sorghum reference genome, using Persephone (https://persephonesoft.com/). The AP13 × VS16 map consists of 946 SNP and SSR markers covering 2,183 cM, and the SG2 × SG1 map contains 562 SNP and SSR markers spanning 2,036 cM.

### 2.5 QTL mapping

Detection of QTL in both species was performed using MapQTL 5 (Van Ooijen, 2004) and using mean FS*n*DOY or heading dates for each *Miscanthus* or switchgrass genotype respectively. Following initial runs using the Interval Mapping method, markers with the highest logarithm of odds (LOD) score were selected and used as initial cofactors in Multiple QTL Model (MQM) mapping analyses. The MQM analyses were reiterated by adding or adjusting one cofactor each run. Cofactors were verified by backward elimination using ‘Automatic Selection of Cofactors’ within the software (Van Ooijen, 2004).

Due to the large number of markers in the outcrossing pseudo‐testcross populations, we used two approaches to reduce prohibitively lengthy MQM run times without substantially sacrificing resolution and power to detect QTL. First, in an exploratory analysis, we recoded genotypes from Cross Pollinator (CP), where parental lines are heterozygous with unknown linkage phases to double haploid genotypes. Next, we split the consensus map into maternal and paternal maps, concatenated these maps and performed an MQM scan on the resulting concatenated map, which excluded fully informative hk x hk markers (i.e. when markers are heterozygous in both parents).

Once potentially significant QTL were identified using this exploratory analysis, we selected cofactors and repeated the MQM analysis using CP genotype codes and all marker types. We then performed MQM runs using CP genotype codes but trimmed the number of markers used to achieve densities of one marker every 1, 2, 3, 4 or 5 cM. These analyses were done in an iterative, stepwise fashion, and the density of markers was chosen based on the number of cofactors used. The final QTL and cofactors chosen were then visualized using GoldenHelix SVS software (http://goldenhelix.com/). Once the MQM analyses produced the choice of a final model, additive (a) and dominant (d) effects were estimated for each significant QTL (Data S1). The ratio of dominant over additive effects was used as an indication of QTL mode of action (Hua et al., 2003). We also calculated the proportion of variance explained (PVE) by each QTL, acknowledging that Beavis effects likely resulted in substantially inflated effect size estimates (Beavis, 1998).

### 2.6 Reference mapping of QTL

Marker positions were established in *Miscanthus* (*M. sinensis* v7.0) and switchgrass (*P. virgatum* v1.1) using NCBI BLASTn v2.6.0 (Altschul et al., 1990) alignment to reference genome assemblies downloaded from Phytozome12 (Goodstein et al., 2012). The *M. sinensis* assembly is 2.08Gb in length, of which 1.9 Gb (90%) is in 19 chromosomes that contain 91% of the annotated genes, whereas 68% of the *P. virgatum* assembly is in 18 chromosomes, named 1–9 (a or b) in accordance with the reference genome of the closely related foxtail millet (*Setaria italica*). Marker flanking sequences were 30–60 bases in length for the *M. sinensis* map and 500 bases for *P. virgatum*. Taking into account allopolyploidy in the *Miscanthus* genus (Kim et al., 2014) and the likelihood of chromosome‐level homology, BLAST hits of marker sequences were stringently filtered to a maximum of one gap and three SNPs. Syntenic blocks were then identified using BLASTN using criteria of alignments of lengths greater than 100 bp and e‐values greater than or equal to 0.00005. Synteny graphs were generated using Circos plot (Krzywinski et al., 2009).

### 2.7 Candidate gene identification

Gene Ontology (GO) annotation was performed on candidate genes in the region of peak QTL, comprising top scoring markers, using the EggNOG orthologous group database (Huerta‐Cepas et al., 2016) v4.5.1. Literature searches were carried out for genes with relevant GO annotations.

## 3 RESULTS

### 3.1 Miscanthus flowering time diversity

Table S2 presents a summary of the average DOY different flowering stages which occurred for the parental accessions Mb111 and Mb121, and the range of DOY values among progeny. Transgressive segregation among progeny resulted in panicle emergence FS2 values ranging from 188 to 232 days, whereas average DOY of FS2 in the parental accessions Mb111 and Mb121 were 201 and 220 respectively. A similar degree of transgressive segregation was observed for all flowering stages. Consecutive flowering stages (e.g. FS3 and FS4) were strongly correlated, but correlations were weaker between more temporally distant stages such as FS1 and AN (Figure S1). Comparing parental accessions in different years, flowering stages were significantly different (ANOVA *p* < .05), with the exception of FS4, where the difference between Mb111 and Mb121 was not statistically significant (data not shown).

While the parental effects remained consistent during all flowering stages, the year effect tended to change significantly towards earlier flowering time in 2011. This could be attributed to the crop's growth maturity that led to an earlier flowering time in year three relative to years one or two. However, we observed significant positive correlations between corresponding flowering stages in different years. Similarly strong correlations were reported in 459 *M. sinensis* accessions, in different years (Zhao et al., 2013).

### 3.2 Switchgrass heading date diversity

In Ardmore and Burneyville, both populations (SG2 × SG1 and AP13 × VS16) exhibited a shorter duration of heading (from the first to the last observations) in 2010 compared to 2009 (Figure S2; Tables S3 and S4). Heading duration in Ardmore and Burneyville for SG2xSG1 progeny in 2009 extended over 58.7 and 56 days, respectively, and 28 and 26.7, respectively in 2010 (Table S3). Likewise, heading periods in the AP13 × VS16 population were 71.7 and 62.7 days in 2009, and 29.2 and 28.8 days in 2010, in Ardmore and Burneyville, respectively (Table S4). However, at each location significant correlations were observed between the heading dates recorded during the 2 years. For instance, among the SG2 × SG1 population, correlations of 0.74 and 0.59 (*p* < .001) were observed at Ardmore and Burneyville respectively.

The mean DOY of heading date in parental lines SG1 and SG2 were 168.5 and 169.5 in Ardmore and 169.3 and 176.0 days in Burneyville respectively. In the progeny, the heading dates ranged from 155.3 to 182.0 days in Ardmore, and 160.3 to 188.3 in Burneyville (Table S3). The average DOY of heading date in 2010 for parental lines VS16 and AP13 was 151.3 and 183.1 in Ardmore and 154.6 and 179.9 in Burneyville respectively. The heading date DOY in their progeny ranged from 151.8 to 183 days in the same year with no significant difference between the locations (Table S4). However, location had significant (*p* < .05) effect on heading dates of the SG2 × SG1 mapping population and their parental lines. On average, plants in Ardmore started heading 3.5 days earlier than in Burneyville. Unlike the AP13 × VS16 population, progeny of SG2xSG1 expressed transgressive segregation at both locations.

### 3.3 Identification of QTL for flowering time in *Miscanthus*

**FIGURE 1:**
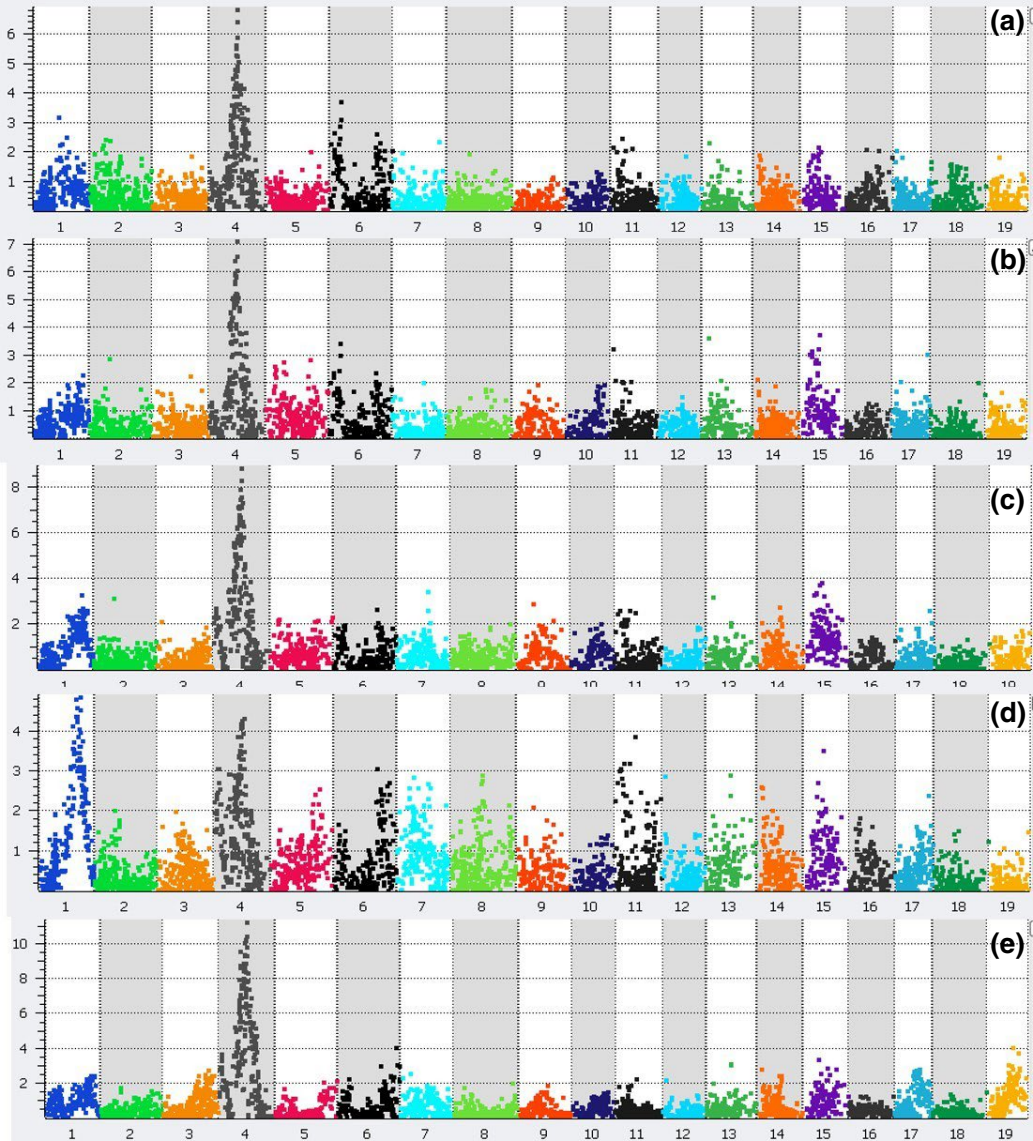
*Miscanthus* flowering time quantitative trait loci mapping. Plots are representative of the whole *Miscanthus* genome and position of single nucleotide polymorphic markers with time of (a) flag leaf emergence, (b) panicle emergence, (c) panicle emergence on >50% of stems, (d) panicle on >80% of stems and (e) onset of anthesis. The *Y*‐axis represents –log10 (*p* values)

A LOD score of greater than 3.5 was considered significant, based on permutation tests, thus a total of 24 QTL were detected across the five flowering stages in *Miscanthus* (Figure 1; Table S5; Figures S3–S7). All five flowering stages showed a strong or very strong QTL in *M. sinensis* LG 4 between 58 and 70 cM (Figure 1). In this region, the QTL for anthesis (*qAN2*) had an LOD score of 15.1–17.03, and the PVE was 29% (Table 1). The data for these QTL suggest *qFS2‐A* has an over dominant effect, while the other colocalized QTL in this region expressed strong additive effects. In our study, marker M00780 was either the top scoring marker for the QTL or among the most significant markers.

**TABLE 1:** Colocalized quantitative trait loci (QTL) based on linkage group and genome‐wide logarithm of odds (LOD) significance for different stages of flowering in *Miscanthus sinensis* (for brevity, negative additive effect relates to Mb111 and positive to Mb121)

QTL	Linkage group	Position (cM)	Peak marker	LOD	PVE^a^ (%)	Additive effect (a)	Dominant effect (d)	QTL mode d/|a|
qFS1‐A	04	62.3–70.65	M04969	8.75	15.53	3.8	−1.49	−0.39
qFS2‐A	04	58.07–70.04	M00780	7.79	11.25	−0.76	−4.33	−5.73
qFS3‐A	04	66.01–70.04	M00780	10.96	19.45	4.4	−5.16	−1.17
qFS4‐B	04	62.17–66.01	M01749	4.37	7.9	2.38	−2.99	−1.26
qAN2	04	66.01–70.04	M00780	17.03	29	4.85	−1.37	−0.28

^a^ Proportion of variance explained (PVE) is acknowledged as a naïve estimate (see Section 2).

Out of 24 significant QTL in *Miscanthus* only four were the product of unexplained genetic interactions, while the additive effect was the prevalent genetic component for the remaining QTL. This points to the potential for manipulating flowering time in *Miscanthus* using marker M00780 through direct selection of parental lines or progeny in segregating populations.

### 3.4 Identification of QTL for heading date in switchgrass

**FIGURE 2:**
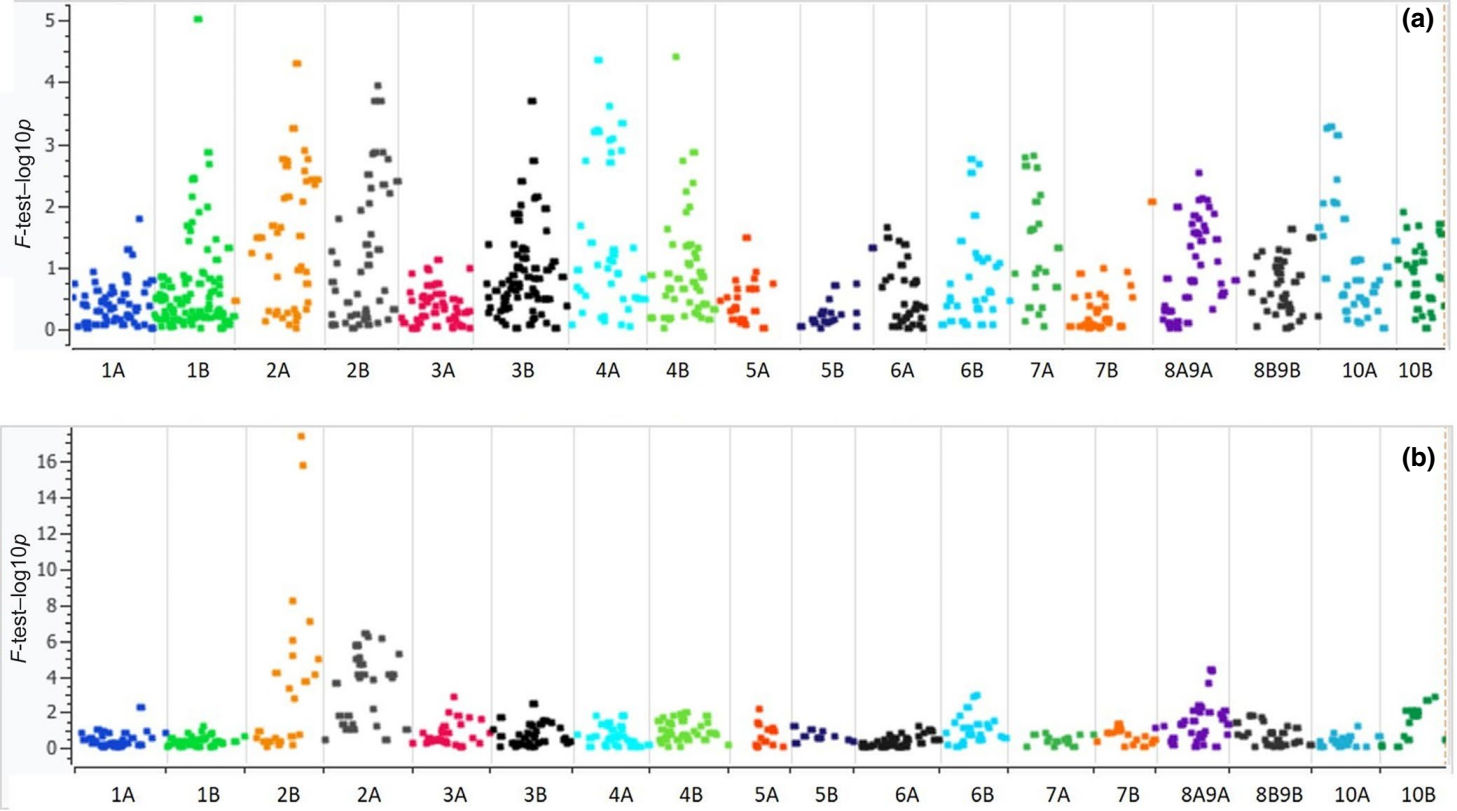
Switchgrass heading date quantitative trait loci mapping. Plots are representative of the whole switchgrass genome and position of single nucleotide polymorphic markers for (a) AP13xVS16 and (b) SG2xSG1. The *X* axis is the switchgrass linkage groups, which are based on sorghum synteny. The *Y*‐axis represents –log10 (*p* values)

Seven QTL were identified for heading date in population AP13 × VS16 and four QTL in population SG2 × SG1 (Figures S8 and S9; Figure 2; Table 2). The QTL landscape in the upland (northern) and lowland (southern) ecotype cross, AP13 × VS16, had clear similarities with the all southern SG2 × SG1 progeny. Of particular significance is the *qPvHD1c*, a SG2 × SG1 QTL with major effect (LOD 21.79) at 86 cM on LG ‘L2B’ that explains 26.9% of phenotypic variation, though this estimate does not account for Beavis effects (see Section 2). Corresponding to *qPvHD1c* are the two QTL at similar positions in the AP13 × VS16 cross; *qPvHD1a* at 87 cM in ‘L2A’ and *qPvHD1b* at 84 cM on ‘L2B’. Both crosses have similarly sized QTL in LG 6B and 8A/9A, although the current maps did not show these loci as very close. There are also differences between the two crosses; the strongest QTL in AP13 × VS16 is *qPvHD3* (LOD 6.8) at 44 cM in LG 4A, but a corresponding QTL was not found in SG2 × SG1.

**TABLE 2:** Switchgrass heading date quantitative trait loci (QTL) based on linkage group and genome‐wide logarithm of odds (LOD) significance (for brevity, negative ‘a’ relates to SG1 or VS16 and positive to SG2 or AP13)

QTL	Population	Linkage group^a^	Foxtail millet chroms	Position (cM)	Peak marker	LOD	PVE^b^ (%)	Additive effect (a)	Dominant effect (d)
*qPvHD1a*	AP13 × VS16	2A	2	86.998	csw165	3.25	2.4	1.71	0.18
*qPvHD1b*	AP13 × VS16	2B	2	84.488	csw1478	5.66	8.1	−2.81	4.71
*qPvHD2*	AP13 × VS16	3B	5	101.51	csw1955	5.83	6.8	−2.35	−1.53
*qPvHD3*	AP13 × VS16	4A	1	44.225	csw11037	6.81	13.8	1.8	1.7
*qPvHD4a*	AP13 × VS16	6B	7	76.845	csw11172	5.77	8.9	−1.7	0.63
*qPvHD5a*	AP13 × VS16	8A/9A	3	70.53	csw1509	4.55	7.8	0.53	2.86
*qPvHD6*	AP13 × VS16	10A	4	41.302	csw1853	4.36	7	−2.25	0.49
*qPvHD1c*	SG2 × SG1	2B	2	85.993	csw1945	21.79	26.9	−4.87	−1.72
*qPvHD1d*	SG2 × SG1	2A	2	64.637	csw11215	7.43	8.8	1.68	−1.74
*qPvHD4b*	SG2 × SG1	6B	7	57.14	csw2406g	3.55	4.1	−1.8	−0.13
*qPvHD5b*	SG2 × SG1	8A/9A	3	83.351	csw1349	6.7	6.8	0.8	−1.06

^a^ Linkage groups of switchgrass are based on sorghum chromosomes.

^b^ Proportion of variance explained (PVE) is acknowledged as a naïve estimate (see Section 2).

### 3.5 QTL synteny in *Miscanthus* and switchgrass

Due to the significance of QTLs on *Miscanthus* LG04, markers within 58–70 cM were chosen to search for candidate genes. Stringent BLAST analysis of flanking sequences of these markers to chromosomal homologues resulted in hits on LG 4 between 74.2 and 85.2 Mb. Furthermore, comparative mapping revealed that the two QTL on LG2A and LG2B in both populations of switchgrass and the five colocalized *Miscanthus* QTL on LG04 were syntenic QTL. Additionally, these QTL corresponded to same genome region on chromosome 2 in sorghum (Table S6), suggesting the underlying loci may play a key role in flowering time/heading date of both species.

### 3.6 Identification of candidate genes

The *Miscanthus* LG04 QTL spans 74.2–85.2 Mb and 448 genes, which make up 11% of the 4,097 putative genes that have been annotated on this chromosome. In the QTL region, 377 genes had predicted orthologues by EggNOG (Huerta‐Cepas et al., 2016). Using a literature search, we identified 11 genes in this region with GO terms associated with regulation of flowering time (Table 3). These included a *Squamosa promoter‐binding protein‐like* (*SPL*) transcription factor (Misin04G229700) within 0.8 Mb of the *qFS1* peak marker, a MADS‐box transcription factor *SEPELLATA2* (Misin04G240700) less than 1 Mb from top scoring QTL markers and a gibberellin‐responsive bHLH137 transcription factor (Misin04G243500) within 0.4 Mb of the flowering time peak markers.

**TABLE 3:** Candidate genes under the *Miscanthus sinensis* LG04 QTL

Gene/marker	Ref. Pos. (Mb)	GO term	Gene name
**M62525**	74.2		
Misin04G201400	74.5	FLOWER	*GAMETE EXPRESSED 1*
Misin04G207500	76.4	GBRESPOND	Alpha‐amyl C2
Misin04G212600	77.5	FLOWER	glucose‐6‐phosphate isomerase 1, chloroplastic‐like
**M01749**	80.5		
**M04969**	81.3		
Misin04G227500	81.6	FLOWER	serine/threonine‐protein kinase UCN‐like
Misin04G229700	82.1	FLOWER	Squamosa promoter binding protein‐like (SPL/SBP domain) transcription factor family protein
**csw165**	82.3		
Misin04G230700	82.3	FLOWER	splicing factor U2af small subunit A‐like
Misin04G233100	82.7	FLOWER	tetraketide alpha pyrone reductase 1 dihydroflavonol‐4‐reductase
Misin04G238500	83.9	FLOWER	3‐deoxy‐mannooctulosonate cytidylyl‐transferase
Misin04G240200	84.2	FLOWER	Meiosis‐specific protein PAIR2
Misin04G240700	84.3	FLOWER	Developmental protein *SEPALLATA2* MADS‐box transcription factor 8
Misin04G243500	84.8	GBRESPOND	Transcription factor bHLH137
**M00780**	85.2		
**M06653**	87.9		

SNP markers are shown in bold, and show the position of candidate genes relative to the markers.

The switchgrass peak marker, csw165, for *qPvHD1a* mapped to about 100 kb from *PvSPL (Ba01711)* on LG 2A (Table 4) and within 200 kb of its orthologue Misin04G240700 in the *Miscanthus sinensis* genome. On the other hand, csw1478, the peak marker for *qPvHD1b* on LG2B in the AP13xVS16 population, mapped to 99.0 Mb on *Miscanthus* LG04.

**TABLE 4:** Conserved synteny of *Miscanthus sinensis* LG04 QTL cluster candidates in *Panicum virgatum* linkage group 2A, showing gene identifiers and positions in each reference and a common gene descriptor

*M. sinensis*	LG04 locus (Mb)	Gene description	*P. virgatum*	LG2A locus (Mb)
Misin04G212600	77.5	glucose‐6phosphateisomerase1, chloroplastic‐like	Ba01978.1	25.6
Misin04G227500	81.6	serine/threonine protein kinase UCN‐like	Ba01728.1	21.8
Misin04G229700	82.1	Squamosa promoter‐binding protein‐like (SPL/SBP)	Ba01711.1	21.7
csw165	82.3			21.6
Misin04G230700	82.3	Splicing factor U2af Small subunit A‐like	Ba01701.3	21.6
Misin04G230700	82.3	Splicing factor U2af Small subunit A‐like	Ba01701.2	21.6
Misin04G233100	82.7	Tetraketide alpha pyrone reductase	Ba01683.1	21.4
Misin04G240200	84.2	Meiosis‐specific protein PAIR2	Ba01532.1	19.9
Misin04G240700	84.3	Developmental protein SEPALLATA2/MADS box TF	Ba01535.1	20.0
Misin04G243500	84.8	TF bHLH137	Ba01554.1	20.2

## 4 DISCUSSION

Mapping populations generated from the perennial grasses *M. sinensis* and switchgrass exhibited a wide variation in flowering time. Despite considerable cross species differences, our data indicate the presence of striking colocalized QTL on *Miscanthus* LG04 and switchgrass LG2, denoting a potentially evolutionarily conserved chromosome block for flowering time in these perennial species. In this region, a major QTL on LG2 is reported to underpin flowering time in a Lowland × Upland switchgrass pseudo‐F_1_ population (Tornqvist et al., 2018). Orthologues of known flowering time genes residing within the QTL identified by Tornqvist et al. include genes involved in: the circadian clock and photoperiod detection (PSEUDO‐RESPONSE REGULATOR 5), transcriptional activation of the floral repressor FLOWERING LOCUS C in the autonomous pathway (SUPPRESSOR OF FRIGIDA 4) and the floral meristem identity gene (APETALA 1). The top scoring markers in the QTL identified in the present study afford greater than twofold additive to dominance effects suggesting these QTL, once confirmed, could be used for implementing MAS in order to, for example, delay flowering in *M. sinensis* or switchgrass to increase biomass yield or improve yield quality.

**FIGURE 3:**
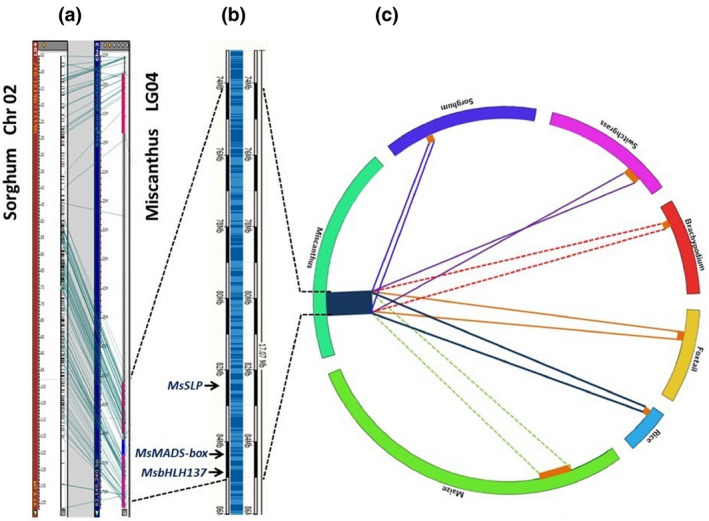
*Miscanthus sinensis* LG04 flowering time quantitative trait loci (QTL). (a) Alignment of the QTL with closely related grass *Sorghum bicolor*. (b) Map position of notable flowering genes on the QTL. (c) Circos plot shows conserved synteny for *M. sinensis* LG04 QTL with closely related grasses. The QTL aligns with *S. bicolor* 2:61971372‐64834820; *Setaria italica* 2:33511249‐37154674; *Zea mays* 7:126570974‐139145447; *Brachypodium distachyon* 4:38082269‐40674492; *Panicum virgatum* 2a: 25824490‐19834290; *Oryza sativa* 9:17459774‐20012773

Our analysis located these QTL in both *Miscanthus* and switchgrass in synteny with 63.1–65.8 Mb on chromosome 2 of sorghum and closely related grasses in the Poaceae family (Figure 3). Several studies in sorghum have shown this chromosomal block accommodates a prominent QTL for flowering time (El Mannai et al., 2012; Srinivas et al., 2009). This flowering QTL was discovered in different sorghum mapping populations, as well as in a Recombinant Inbred Lines population of 96B × IS18551 (Srinivas et al., 2009), and an F_2_ generation of Kikuchi Zairai × SC 112 (El Mannai et al., 2012), which is located in the flanking region of an SSR marker, *Xtxp298* (Srinivas et al., 2009). Furthermore, a study on brassinosteroids in sorghum identified a QTL for flowering time in the flanking region of *Xtxp298*. Subsequently, the authors suggested *BR INSENSITIVE KINASE INHIBITOR 1 (BKI1)* as a candidate gene with pleiotropic effects governing the flowering time and several growth aspects in sorghum (Perez et al., 2014). On the other hand, the *Xtxp298* marker was shown to be tightly associated with a QTL for the stay‐green trait (Galyuon et al., 2016), a characteristic that delays senescence postflowering and could result in greater biomass yield (Clifton‐Brown et al., 2002; Gregersen et al., 2013).

In this context, a *GLUTAMATE S‐SEMIALDEHYDE DEHYDROGENASE* (Sobic.002G215700) was proposed as a candidate gene governing the stay‐green trait in coordination with three other QTL (Johnson et al., 2015). Given the complex nature and independent biological pathways of flowering time and stay‐green traits, the phenotypes are often found to be correlated (Miryeganeh et al., 2018). This was attributed to either phenological synchronization (Miryeganeh et al., 2018) or a linkage disequilibrium block that comprised genes underpinning a stay‐green tendency and day length insensitivity (Thomas & Ougham, 2014).

A large number of genes are involved in the control of flowering, and flowering itself is impacted by diverse environmental and metabolic signalling pathways (Buckler et al., 2009). The age‐related flowering pathway can trigger floral transition if other inducing conditions fail (Teotia & Tang, 2015; Wang et al., 2009). Two miRNAs are critical components of this pathway—the evolutionarily conserved miR156 suppresses flowering by repressing *SQUAMOSA PROMOTER BINDING‐LIKE* (SPL) transcription factors, and flowering activator miR172 (Amasino, 2010; Wang et al., 2009). miR172 promotes flowering by repression of APETALA 2‐like floral transition repressors (Aukerman & Sakai, 2003). The observed declining abundance of miR156 transcripts with age in perennial species (Bergonzi et al., 2013; Wang et al., 2011) is potentially an endogenous cue that stimulates flowering (Bergonzi et al., 2013). The miR156/SPL transcription factor pathway is an element in floral initiation in *P. virgatum* (Johnson et al., 2017). Overexpression of the maize Corngrass1 (*Cg1*) gene, which encodes miRNA156, delays flowering in maize and prevents flowering in switchgrass (Chuck et al., 2011).

In *Miscanthus*, the identified QTL includes two genes previously identified as being part of the age‐related flowering pathway. The *SQUAMOSA PROMOTER BINDING PROTEIN‐LIKE (SPL/SBP* domain) family protein (Misin04G229700) is within 0.8 Mb of the peak marker of *Miscanthus qFS1‐B* and its orthologue is within 100kb of switchgrass *qPvHD1a*. This conserved transcription factor in the age‐dependent pathway (Shalom et al., 2015; Wang et al., 2009; Wu et al., 2009) is known to modulate flowering in switchgrass (Baxter et al., 2018; Johnson et al., 2017).

The MADS‐box transcription factor *SEPELLATA2* (Misin04G240700) is also part of the age‐dependent flowering pathway (Balanza et al., 2018; Wu et al., 2009). This transcription factor is within 1 Mb of the QTL peaks of *qFS2‐A*, *qFS3‐A* and *qAN2*, and is 91% identical to MADS‐box transcription factor 8 (*OsMADS8*), a member of the SEP3 clade (*AtSEPALLATA*) that controls floral organ specification (Cui et al., 2010). A gibberellin‐responsive bHLH137 transcription factor (Misin04G243500) is also within 0.4 Mb of the QTL peaks of *qFS2‐A*, *qF3‐A* and *qAN2*. A similar gene is a regulator of the anthocyanin pathway in *Gerbera hybrida* (Elomaa et al., 1998).

The remaining QTL contained genes that had previously been characterized for flowering. However, these were generally specific to either *Miscanthus* or switchgrass and were mid‐ to minor‐effect QTL. This may point to the complexity and specificity of the biological pathways governing flowering time in each species.

Despite great ecological and genetic differences between the AP13 × VS16 and SG2 × SG1 populations and the existence of reported significant family × location × year interactions for flowering in switchgrass (Bhandari et al., 2010), notable QTL were identified on comparable LGs, and map positions of significant markers in the two populations were remarkably similar. Expanding markers in these genomic regions would facilitate fine mapping and subsequent identification of markers closely linked to flowering traits.

Although the detection of syntenic flowering QTL across such a broad phylogenetic distance is not unprecedented for the Poaceae (Armstead et al., 2004; Chardon et al., 2004; Mauro‐Herrera et al., 2013), it raises intriguing evolutionary questions. Was there an ancestral QTL that retained its relative importance over millennia of exposure to ionizing radiation? Or did the mutations underlying the QTL evolve in parallel in multiple genera and species? Answering these questions will be difficult without more specific and definitive information about the polymorphisms underlying the QTL, but analysis‐oriented QTL catalogues within and beyond the Saccharineae clade (Zhang et al., 2013), as well as the ongoing larger scale genome‐wide association studies in multiple *Miscanthus* species (Clifton‐Brown et al., 2019), will provide at least initial insights.

## AUTHOR CONTRIBUTION

E.J. managed the BBSRC *Miscanthus* flowering project including mapping family establishment and maintenance, phenotyping and harvesting, data analysis and manuscript preparation and review; R.S. conducted genomics and statistical analysis, interpreted data and contributed to manuscript preparation and review; G.T.S. was involved in *Miscanthus* data analysis and interpretation, as well as in drafting and revising the manuscript; X.‐F.M. conducted linkage mapping and QTL mapping of the three populations, drafted analysing method and revised the manuscript; D.D.S. conducted switchgrass field experiments and collected and analysed data; D.P.S. aligned genetic map markers to the references, identified and GO annotated candidate genes under the QTL marker peaks in both species, and found the literature support for flowering candidates, including the SPL/SBP family protein (Misin04G229700) and *SEPELLATA2* (Misin04G240700). He also contributed to early manuscript drafts; P.R. contributed to conceptualization, managed *Miscanthus* trial harvesting, reviewed and edited manuscript; S.T.J. helped propagate and establish the *Miscanthus* mapping family and conducted phenotyping and harvesting; T.S. managed genotyping of the three populations.

J.C.‐B. led the breeding programme project with DEFRA where Mx2 was made by breeder Charlotte Hayes. He designed the *Miscanthus* field trial, the phenotyping protocols with E.J. and support from IBERS statistician Ruth Sanderson. J.C.‐B. led the GIANT LINK project with R.F. from CERES Inc, which supported R.S., T.S. and X.‐F.M. M.C.S. contributed to the conception and design of switchgrass studies and manuscript revision. I.D. set up a succession of BBSRC projects with J.C.‐B. including the BBSRC flowering grant which supported E.J. and S.T.J. E.J. was managed by K.F., who helped shape the study. I.D. initiated the IBERS‐Nobel Foundation collaboration on flowering QTL in Miscanthus and switchgrass.

All authors reviewed the manuscript.

## Supporting information

Supplementary Material

## Data Availability

The data that support the findings of this study are available in the supplementary material of this article.
