# Supplementary material for: Linkage mapping evidence for a syntenic QTL associated with flowering time in perennial C4 rhizomatous grasses Miscanthus and switchgrass
Source: Glob Change Biol Bioenergy. 2020 Oct 28;13(1):98–111. doi: 10.1111/gcbb.12755 (PMC7756372; doi:10.1111/gcbb.12755)
Supplement: Supplementary file 1 — Supplementary Material [file GCBB-13-98-s001.pptx]

## Slide 1
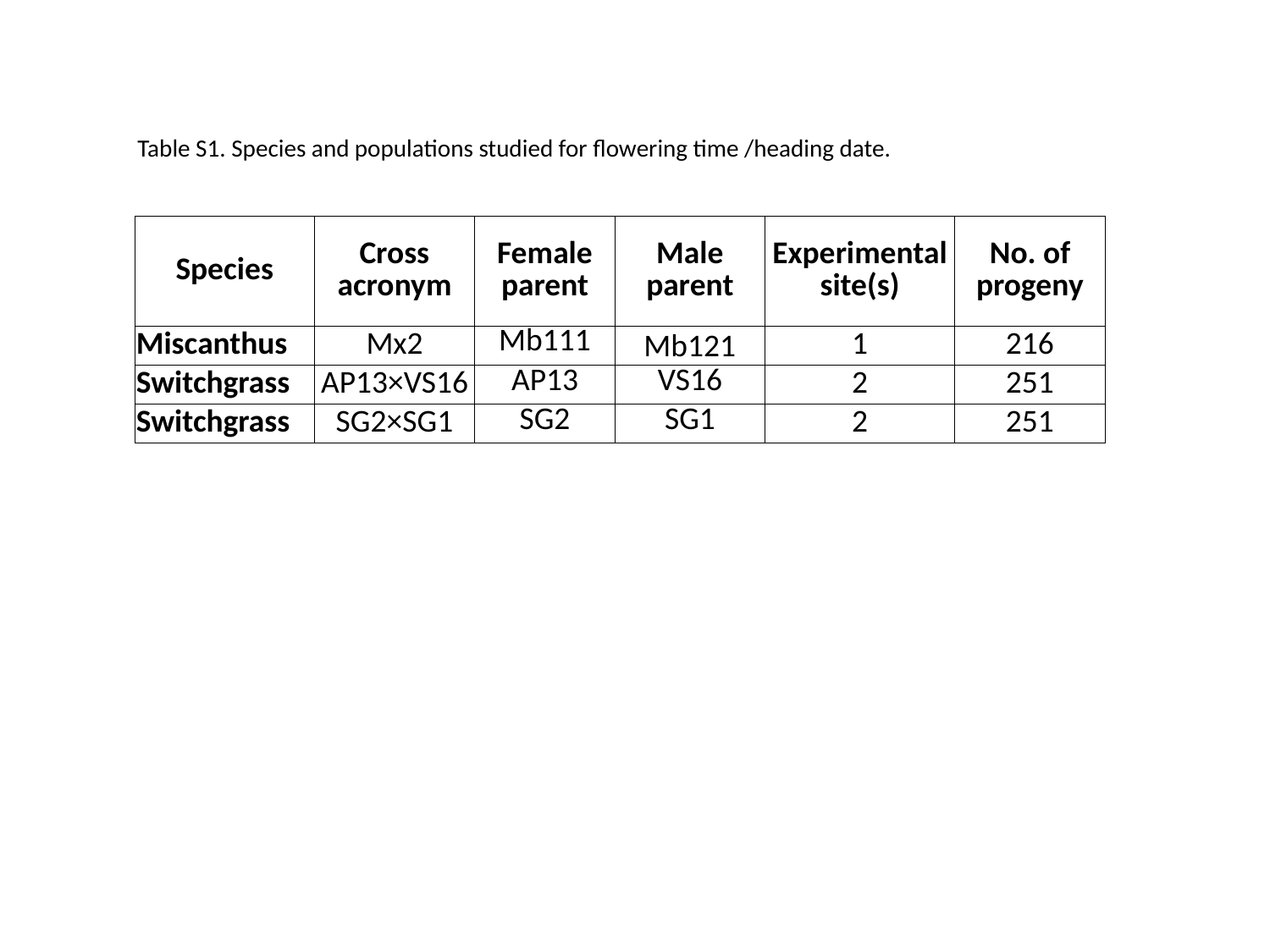

Table S1. Species and populations studied for flowering time /heading date.
| Species | Cross acronym | Female parent | Male parent | Experimental site(s) | No. of progeny |
| --- | --- | --- | --- | --- | --- |
| Miscanthus | Mx2 | Mb111 | Mb121 | 1 | 216 |
| Switchgrass | AP13×VS16 | AP13 | VS16 | 2 | 251 |
| Switchgrass | SG2×SG1 | SG2 | SG1 | 2 | 251 |

## Slide 2
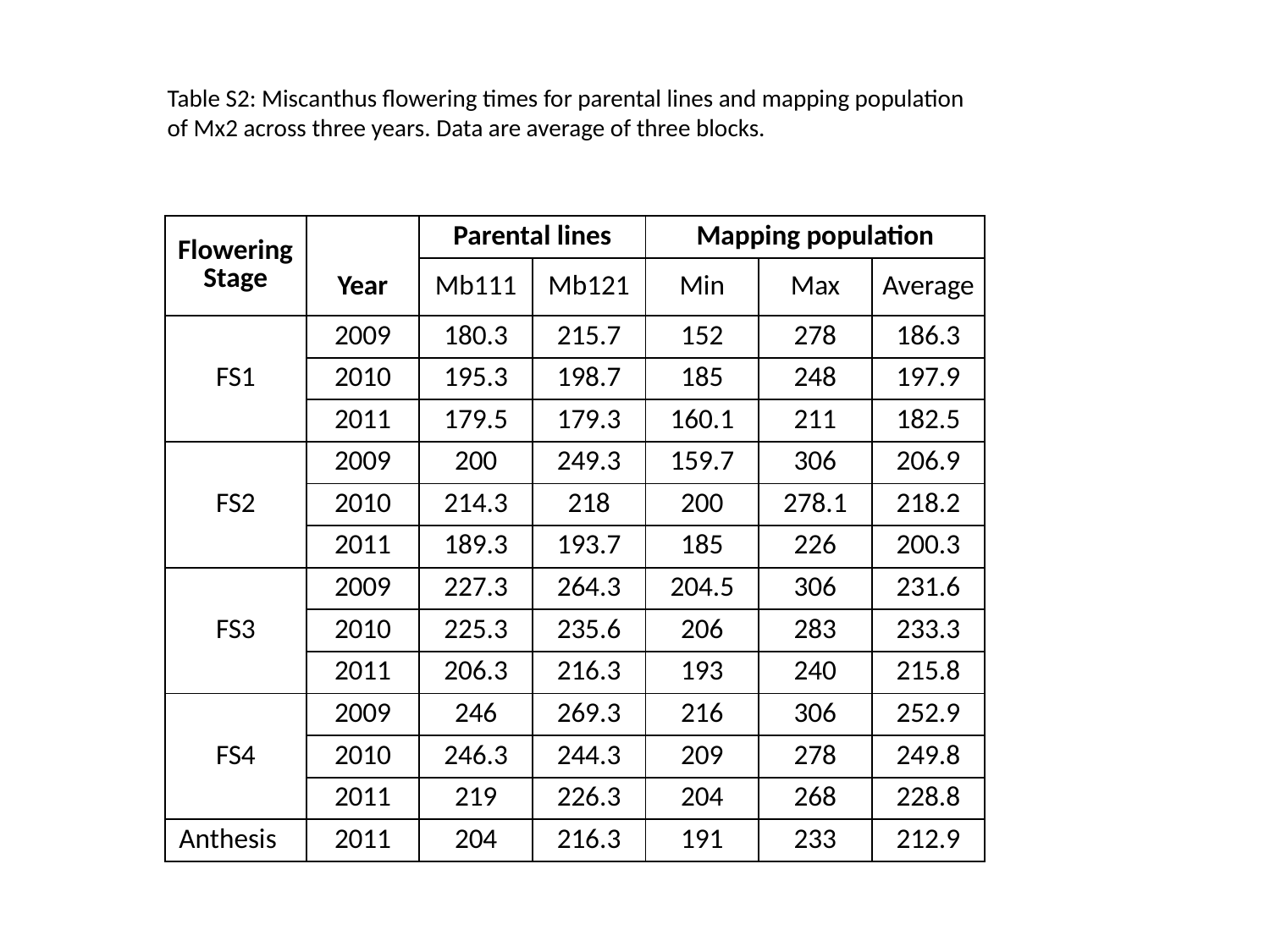

Table S2: Miscanthus flowering times for parental lines and mapping population of Mx2 across three years. Data are average of three blocks.
| Flowering Stage | | Parental lines | | Mapping population | | |
| --- | --- | --- | --- | --- | --- | --- |
| | Year | Mb111 | Mb121 | Min | Max | Average |
| FS1 | 2009 | 180.3 | 215.7 | 152 | 278 | 186.3 |
| | 2010 | 195.3 | 198.7 | 185 | 248 | 197.9 |
| | 2011 | 179.5 | 179.3 | 160.1 | 211 | 182.5 |
| FS2 | 2009 | 200 | 249.3 | 159.7 | 306 | 206.9 |
| | 2010 | 214.3 | 218 | 200 | 278.1 | 218.2 |
| | 2011 | 189.3 | 193.7 | 185 | 226 | 200.3 |
| FS3 | 2009 | 227.3 | 264.3 | 204.5 | 306 | 231.6 |
| | 2010 | 225.3 | 235.6 | 206 | 283 | 233.3 |
| | 2011 | 206.3 | 216.3 | 193 | 240 | 215.8 |
| FS4 | 2009 | 246 | 269.3 | 216 | 306 | 252.9 |
| | 2010 | 246.3 | 244.3 | 209 | 278 | 249.8 |
| | 2011 | 219 | 226.3 | 204 | 268 | 228.8 |
| Anthesis | 2011 | 204 | 216.3 | 191 | 233 | 212.9 |

## Slide 3
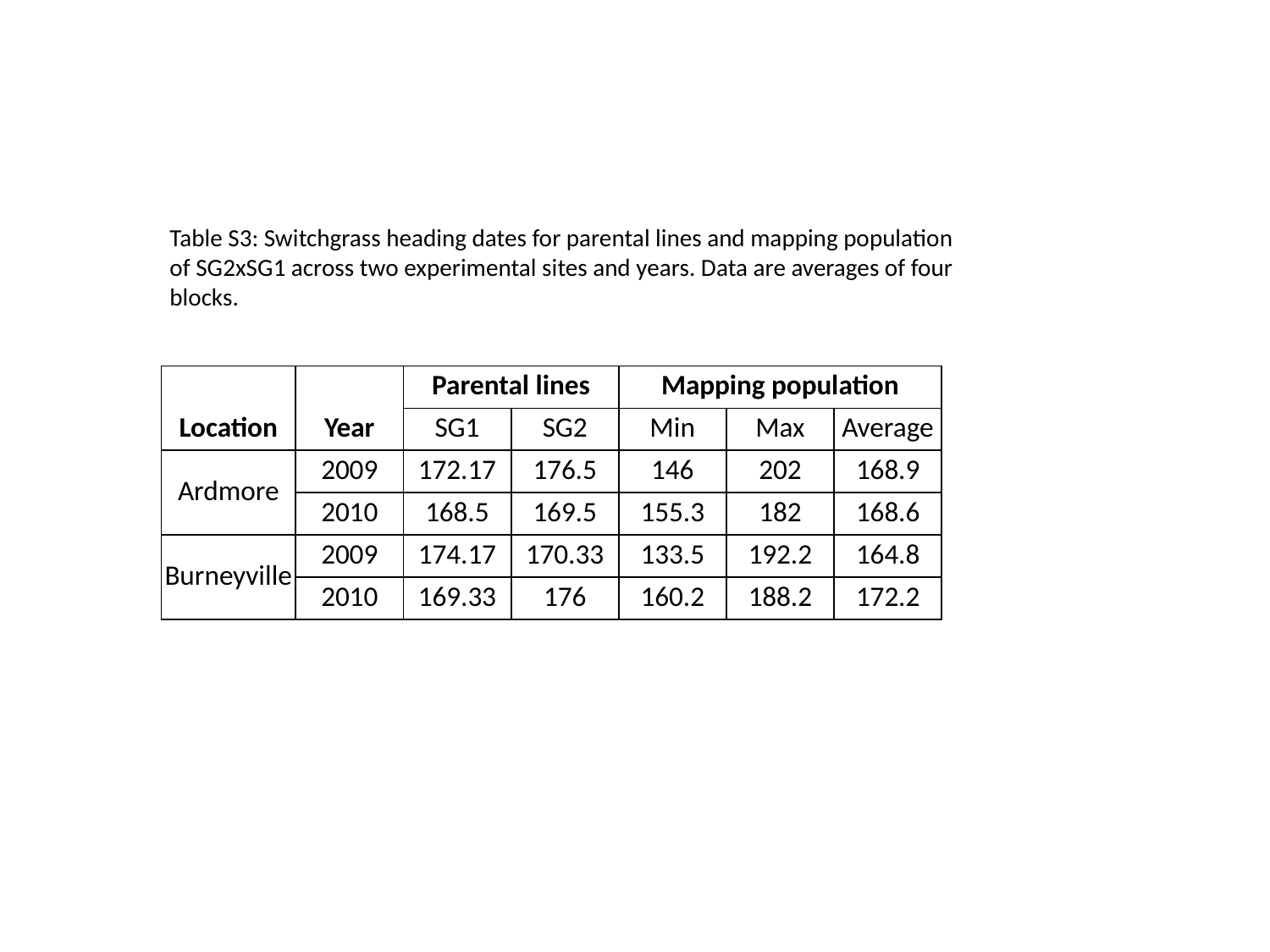

Table S3: Switchgrass heading dates for parental lines and mapping population of SG2xSG1 across two experimental sites and years. Data are averages of four blocks.
| | | Parental lines | | Mapping population | | |
| --- | --- | --- | --- | --- | --- | --- |
| Location | Year | SG1 | SG2 | Min | Max | Average |
| Ardmore | 2009 | 172.17 | 176.5 | 146 | 202 | 168.9 |
| | 2010 | 168.5 | 169.5 | 155.3 | 182 | 168.6 |
| Burneyville | 2009 | 174.17 | 170.33 | 133.5 | 192.2 | 164.8 |
| | 2010 | 169.33 | 176 | 160.2 | 188.2 | 172.2 |

## Slide 4
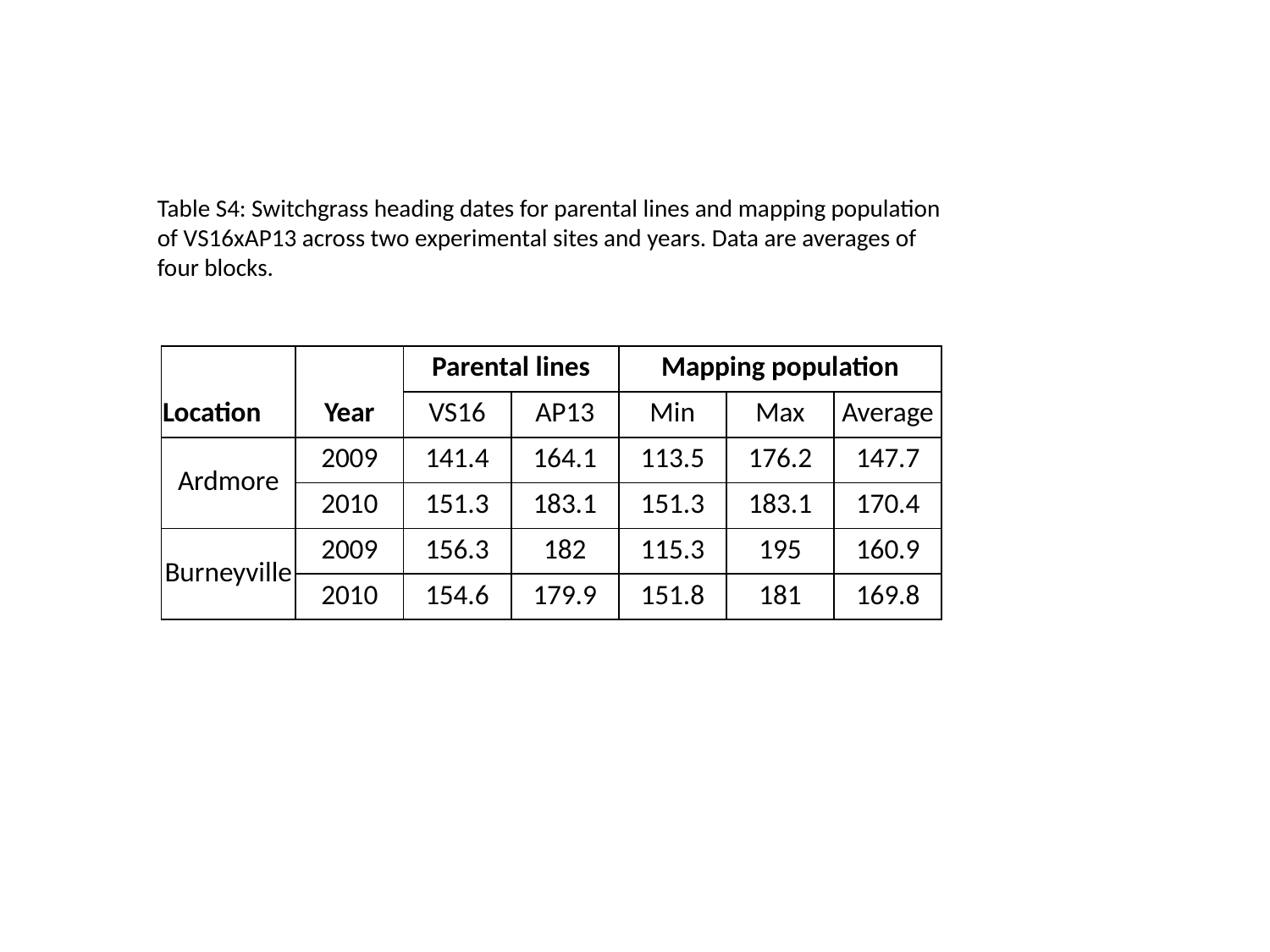

Table S4: Switchgrass heading dates for parental lines and mapping population of VS16xAP13 across two experimental sites and years. Data are averages of four blocks.
| | | Parental lines | | Mapping population | | |
| --- | --- | --- | --- | --- | --- | --- |
| Location | Year | VS16 | AP13 | Min | Max | Average |
| Ardmore | 2009 | 141.4 | 164.1 | 113.5 | 176.2 | 147.7 |
| | 2010 | 151.3 | 183.1 | 151.3 | 183.1 | 170.4 |
| Burneyville | 2009 | 156.3 | 182 | 115.3 | 195 | 160.9 |
| | 2010 | 154.6 | 179.9 | 151.8 | 181 | 169.8 |

## Slide 5
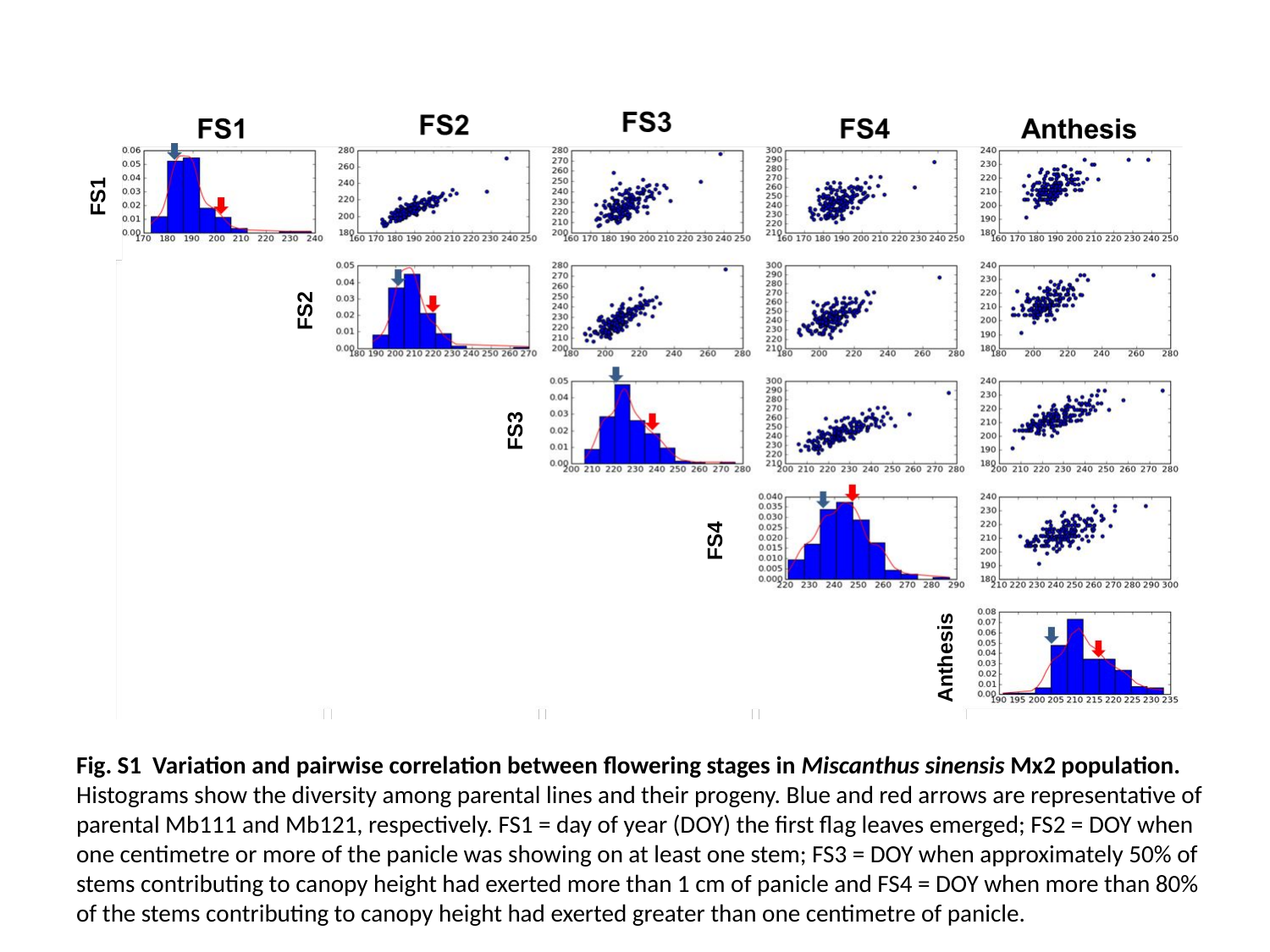

FS1
FS2
FS3
FS4
Anthesis
Fig. S1 Variation and pairwise correlation between flowering stages in Miscanthus sinensis Mx2 population. Histograms show the diversity among parental lines and their progeny. Blue and red arrows are representative of parental Mb111 and Mb121, respectively. FS1 = day of year (DOY) the first flag leaves emerged; FS2 = DOY when one centimetre or more of the panicle was showing on at least one stem; FS3 = DOY when approximately 50% of stems contributing to canopy height had exerted more than 1 cm of panicle and FS4 = DOY when more than 80% of the stems contributing to canopy height had exerted greater than one centimetre of panicle.

## Slide 6
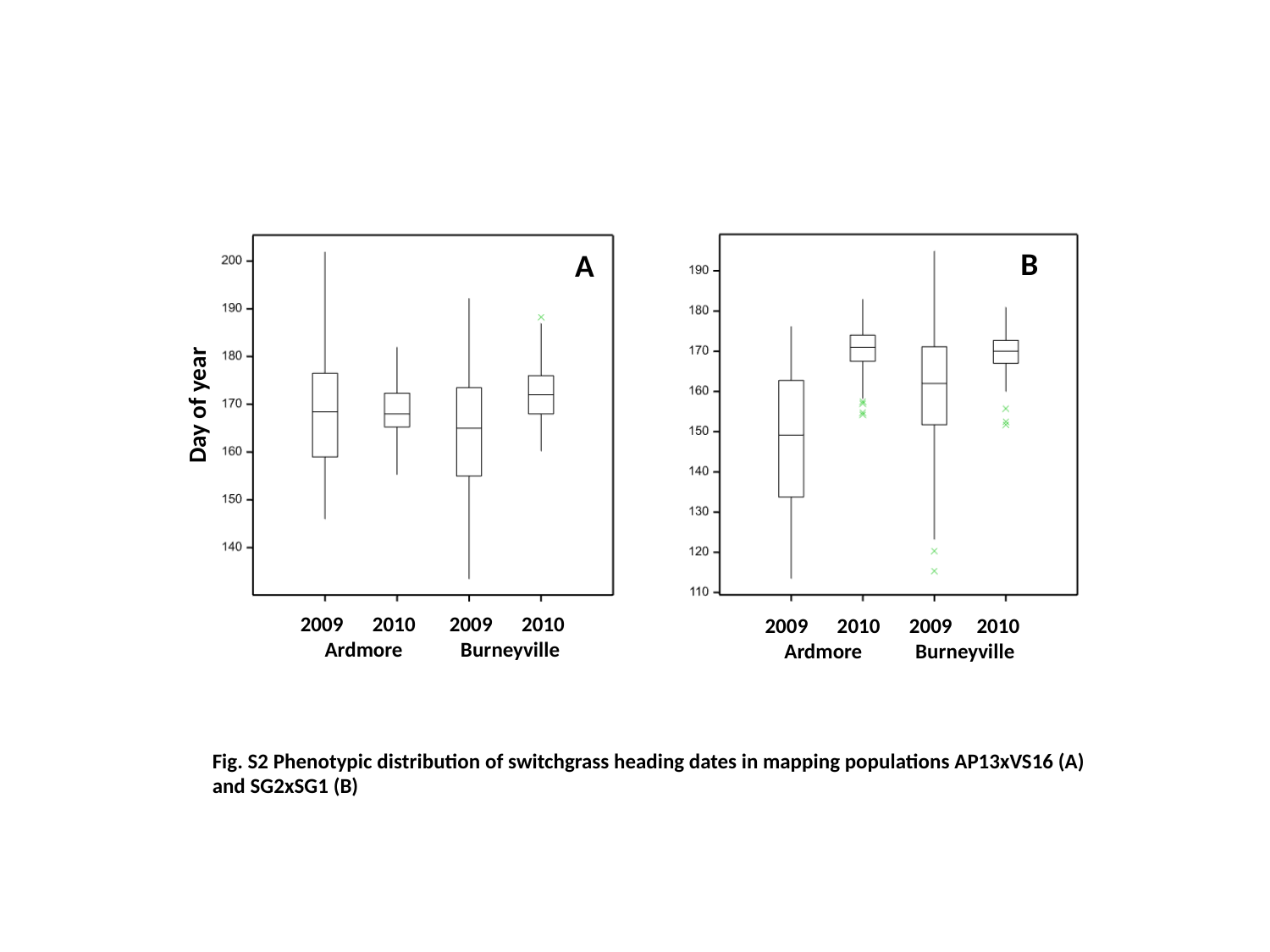

B
 2009 2010 2009 2010
 Ardmore Burneyville
A
 2009 2010 2009 2010
 Ardmore Burneyville
Day of year
Fig. S2 Phenotypic distribution of switchgrass heading dates in mapping populations AP13xVS16 (A) and SG2xSG1 (B)

## Slide 7
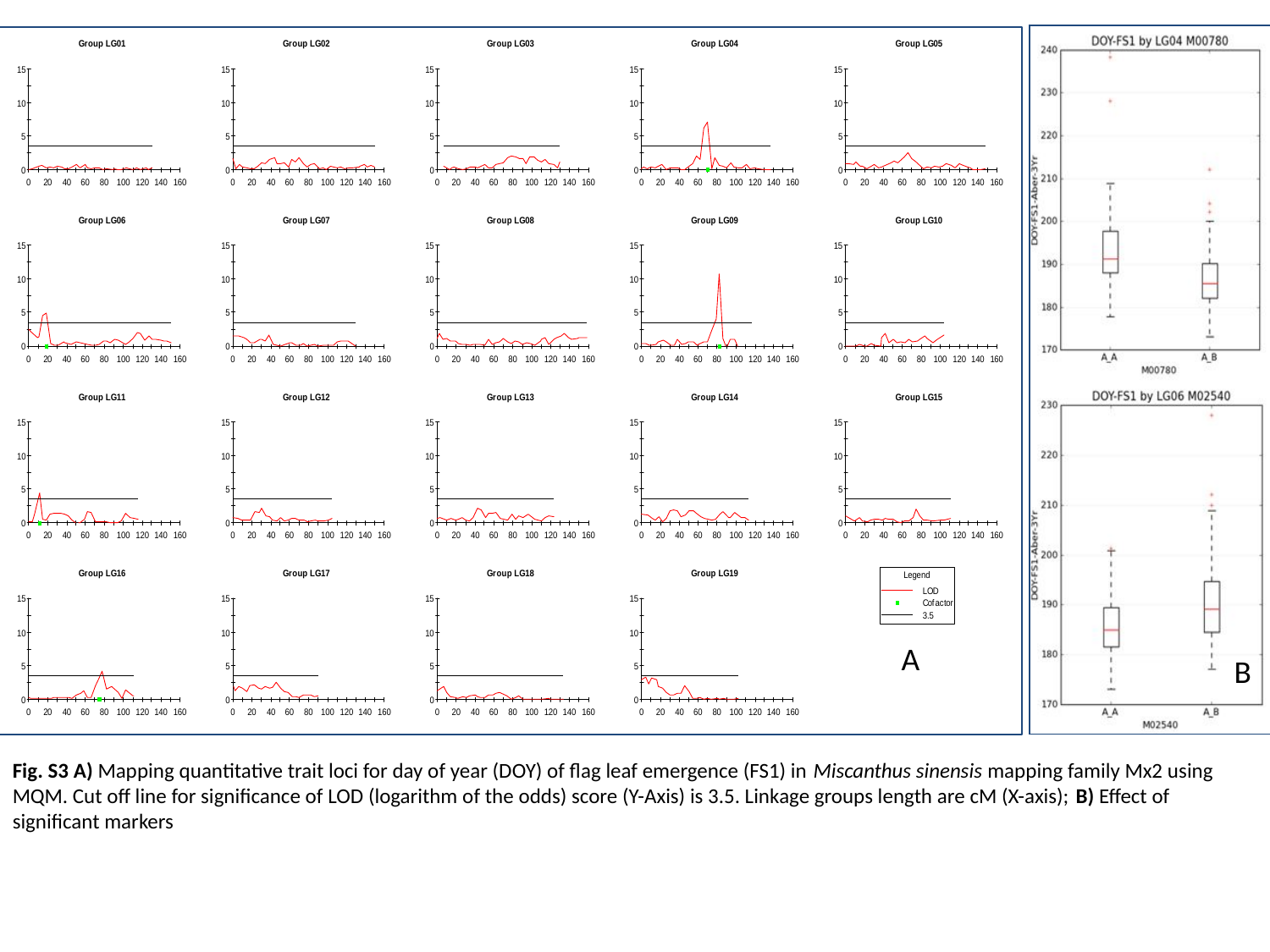

A
B
Fig. S3 A) Mapping quantitative trait loci for day of year (DOY) of flag leaf emergence (FS1) in Miscanthus sinensis mapping family Mx2 using MQM. Cut off line for significance of LOD (logarithm of the odds) score (Y-Axis) is 3.5. Linkage groups length are cM (X-axis); B) Effect of significant markers

## Slide 8
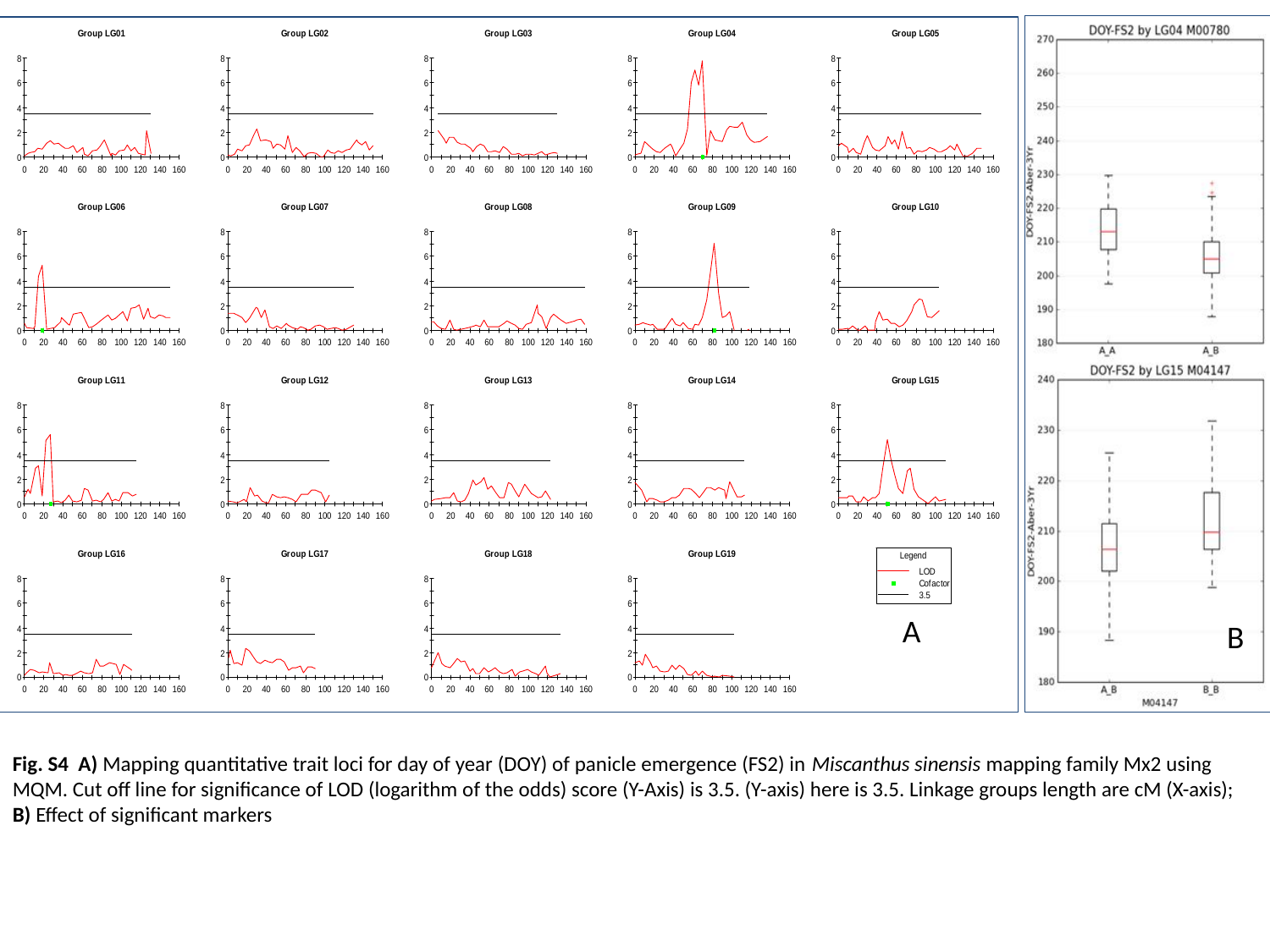

B
A
Fig. S4 A) Mapping quantitative trait loci for day of year (DOY) of panicle emergence (FS2) in Miscanthus sinensis mapping family Mx2 using MQM. Cut off line for significance of LOD (logarithm of the odds) score (Y-Axis) is 3.5. (Y-axis) here is 3.5. Linkage groups length are cM (X-axis); B) Effect of significant markers

## Slide 9
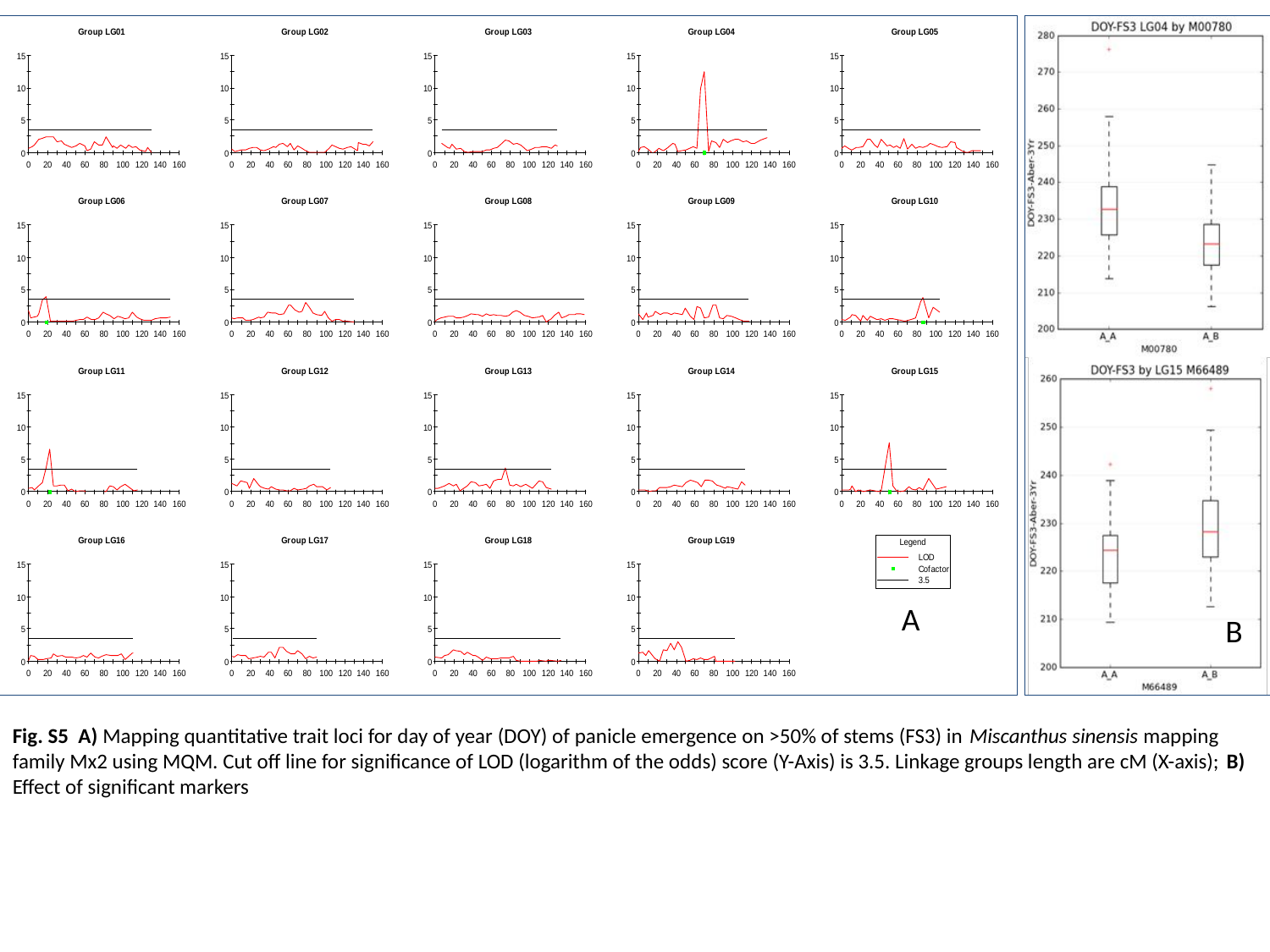

A
B
Fig. S5 A) Mapping quantitative trait loci for day of year (DOY) of panicle emergence on >50% of stems (FS3) in Miscanthus sinensis mapping family Mx2 using MQM. Cut off line for significance of LOD (logarithm of the odds) score (Y-Axis) is 3.5. Linkage groups length are cM (X-axis); B) Effect of significant markers

## Slide 10
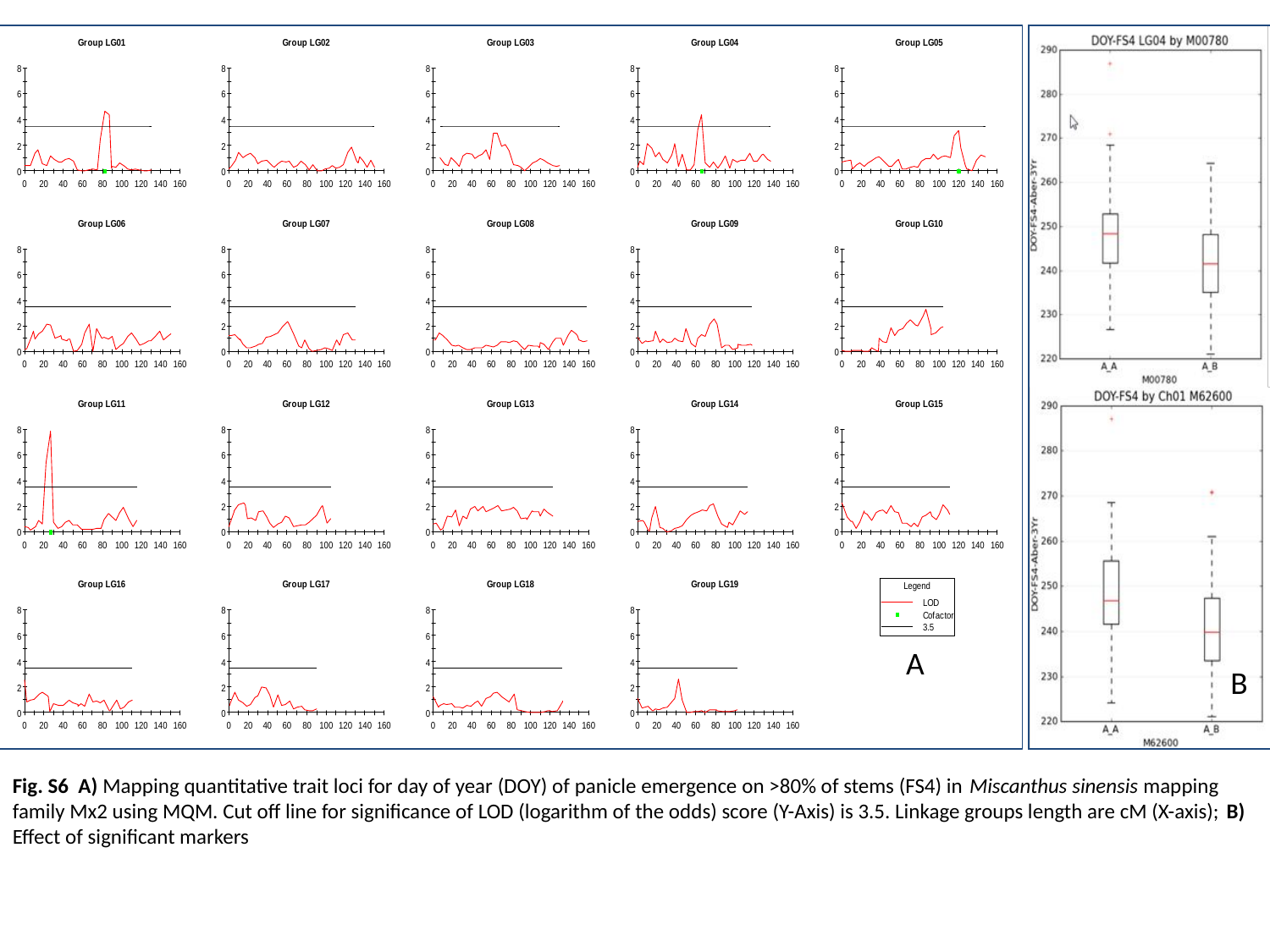

A
B
Fig. S6 A) Mapping quantitative trait loci for day of year (DOY) of panicle emergence on >80% of stems (FS4) in Miscanthus sinensis mapping family Mx2 using MQM. Cut off line for significance of LOD (logarithm of the odds) score (Y-Axis) is 3.5. Linkage groups length are cM (X-axis); B) Effect of significant markers

## Slide 11
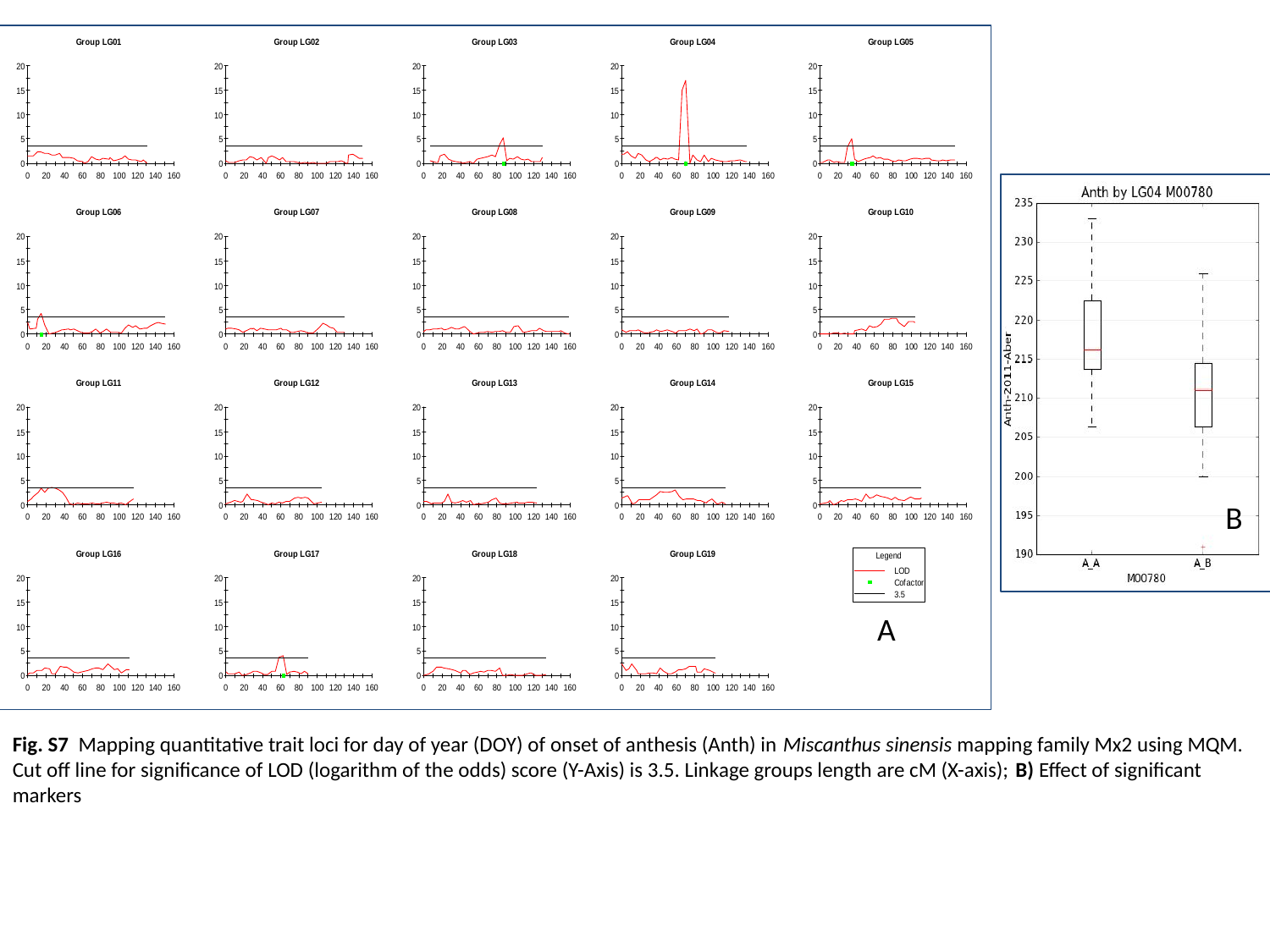

B
A
Fig. S7 Mapping quantitative trait loci for day of year (DOY) of onset of anthesis (Anth) in Miscanthus sinensis mapping family Mx2 using MQM. Cut off line for significance of LOD (logarithm of the odds) score (Y-Axis) is 3.5. Linkage groups length are cM (X-axis); B) Effect of significant markers

## Slide 12
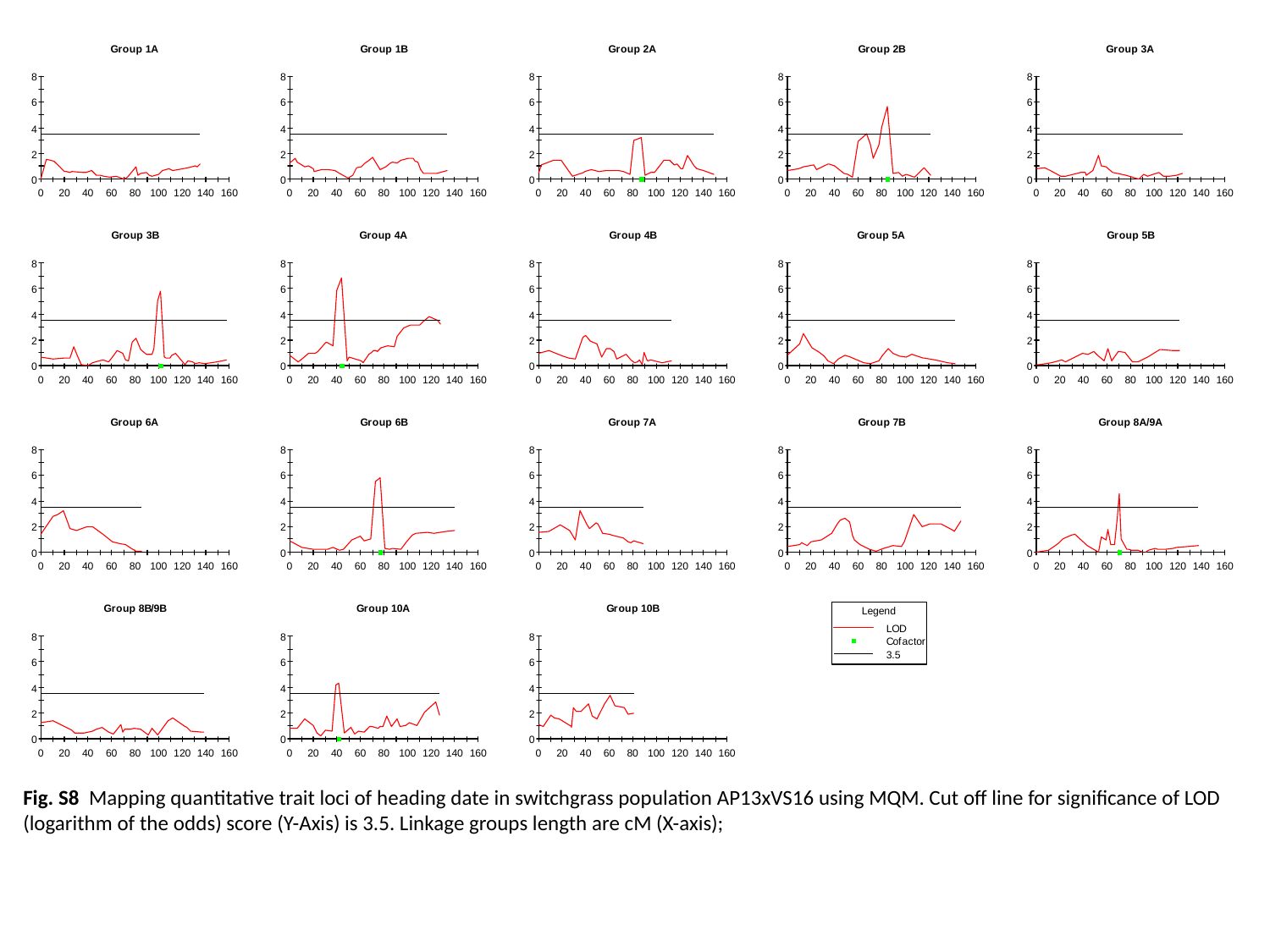

Fig. S8 Mapping quantitative trait loci of heading date in switchgrass population AP13xVS16 using MQM. Cut off line for significance of LOD (logarithm of the odds) score (Y-Axis) is 3.5. Linkage groups length are cM (X-axis);

## Slide 13
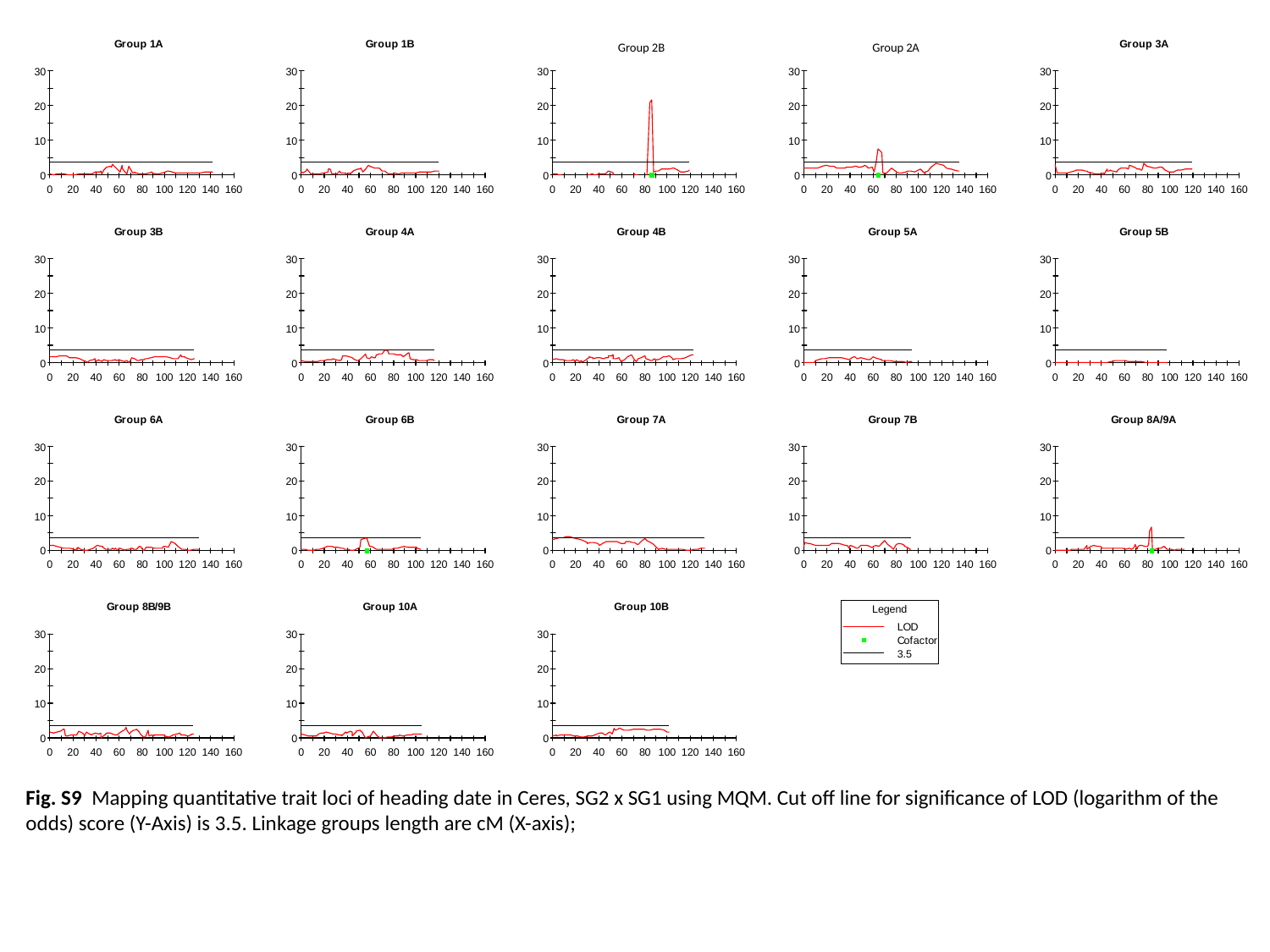

Group 2B
Group 2A
Fig. S9 Mapping quantitative trait loci of heading date in Ceres, SG2 x SG1 using MQM. Cut off line for significance of LOD (logarithm of the odds) score (Y-Axis) is 3.5. Linkage groups length are cM (X-axis);

## Slide 14
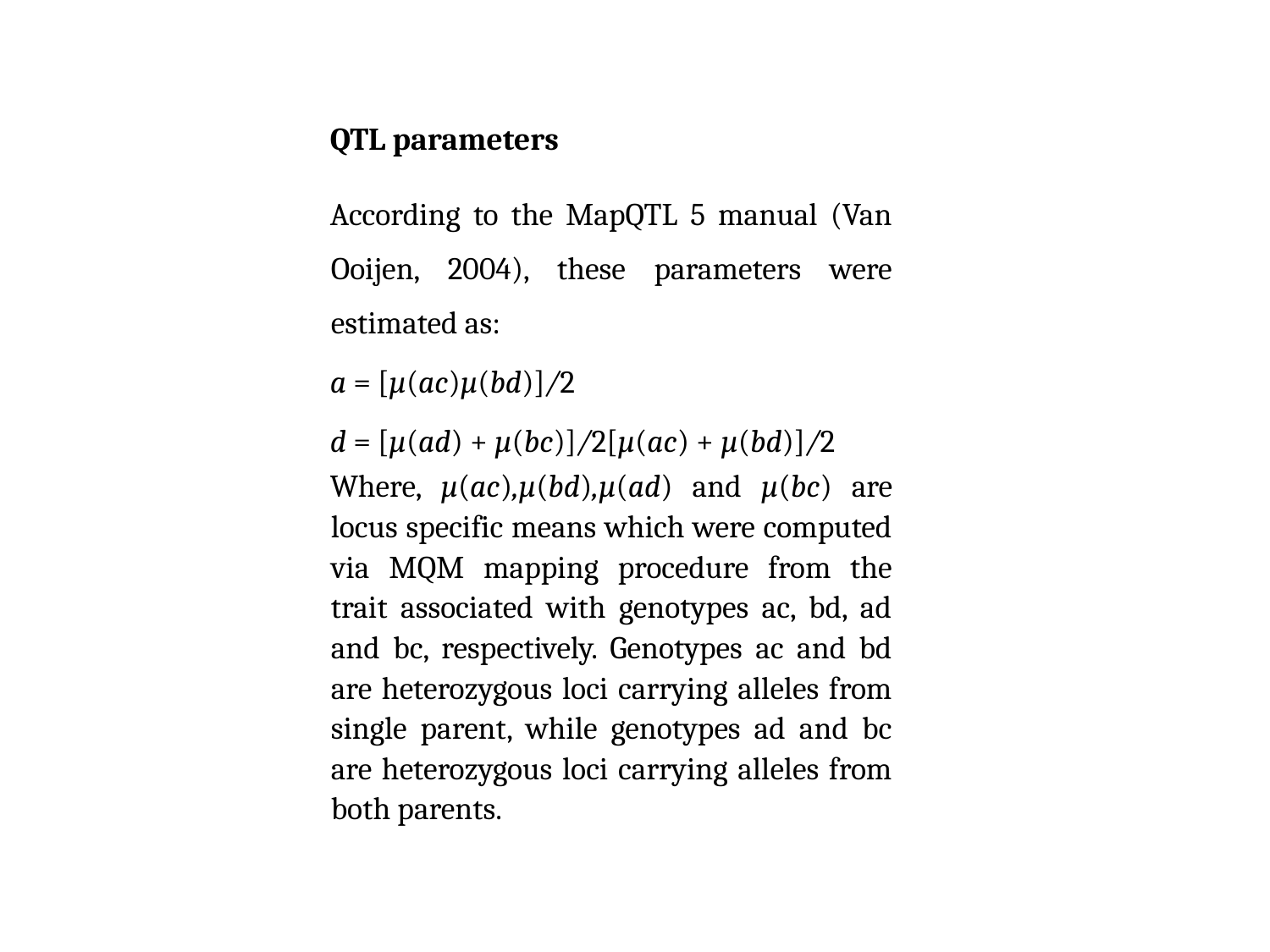

QTL parameters
According to the MapQTL 5 manual (Van Ooijen, 2004), these parameters were estimated as:
a = [µ(ac)µ(bd)]/2
d = [µ(ad) + µ(bc)]/2[µ(ac) + µ(bd)]/2
Where, µ(ac),µ(bd),µ(ad) and µ(bc) are locus specific means which were computed via MQM mapping procedure from the trait associated with genotypes ac, bd, ad and bc, respectively. Genotypes ac and bd are heterozygous loci carrying alleles from single parent, while genotypes ad and bc are heterozygous loci carrying alleles from both parents.

## Slide 15
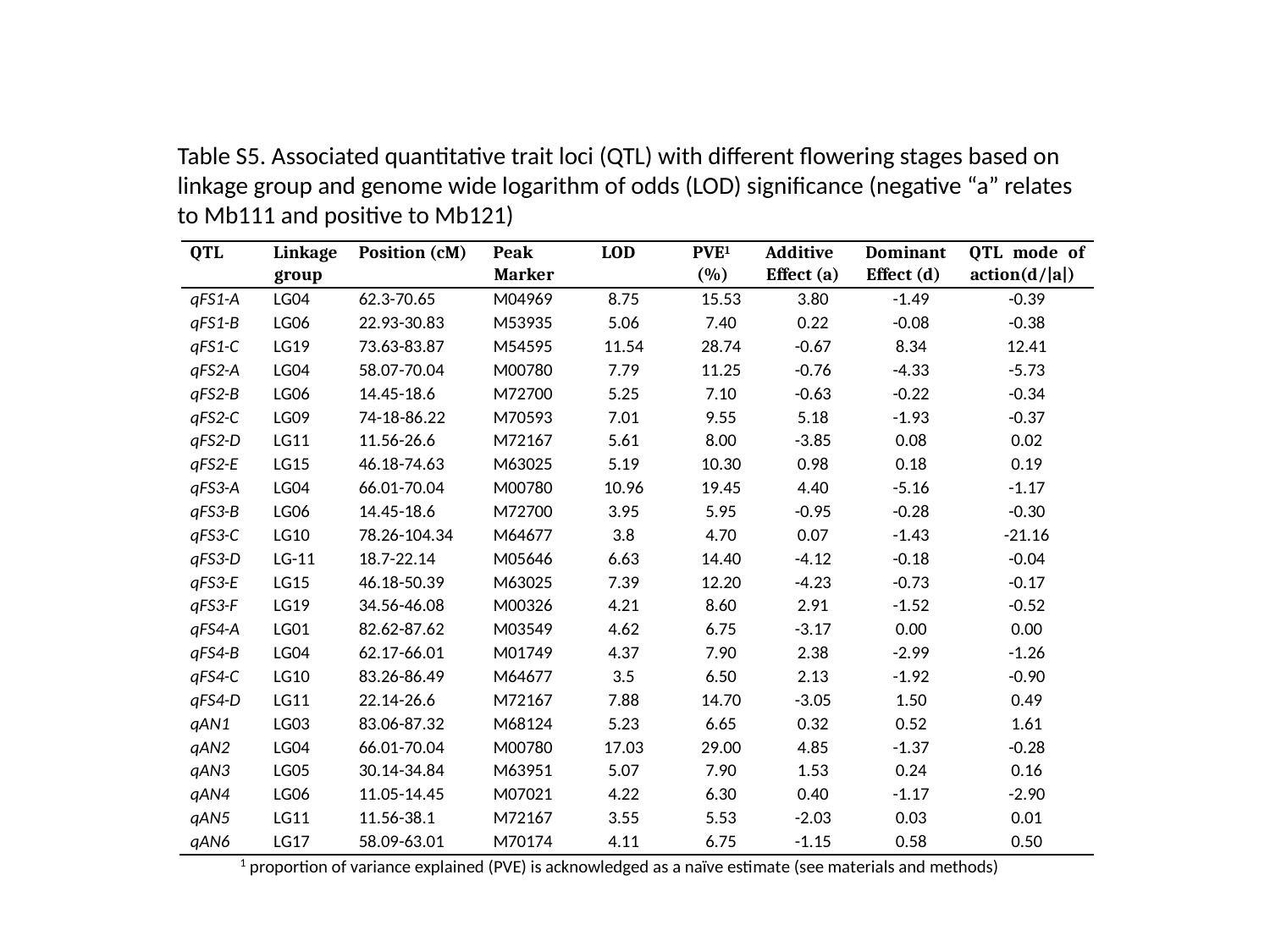

Table S5. Associated quantitative trait loci (QTL) with different flowering stages based on linkage group and genome wide logarithm of odds (LOD) significance (negative “a” relates to Mb111 and positive to Mb121)

## Slide 16
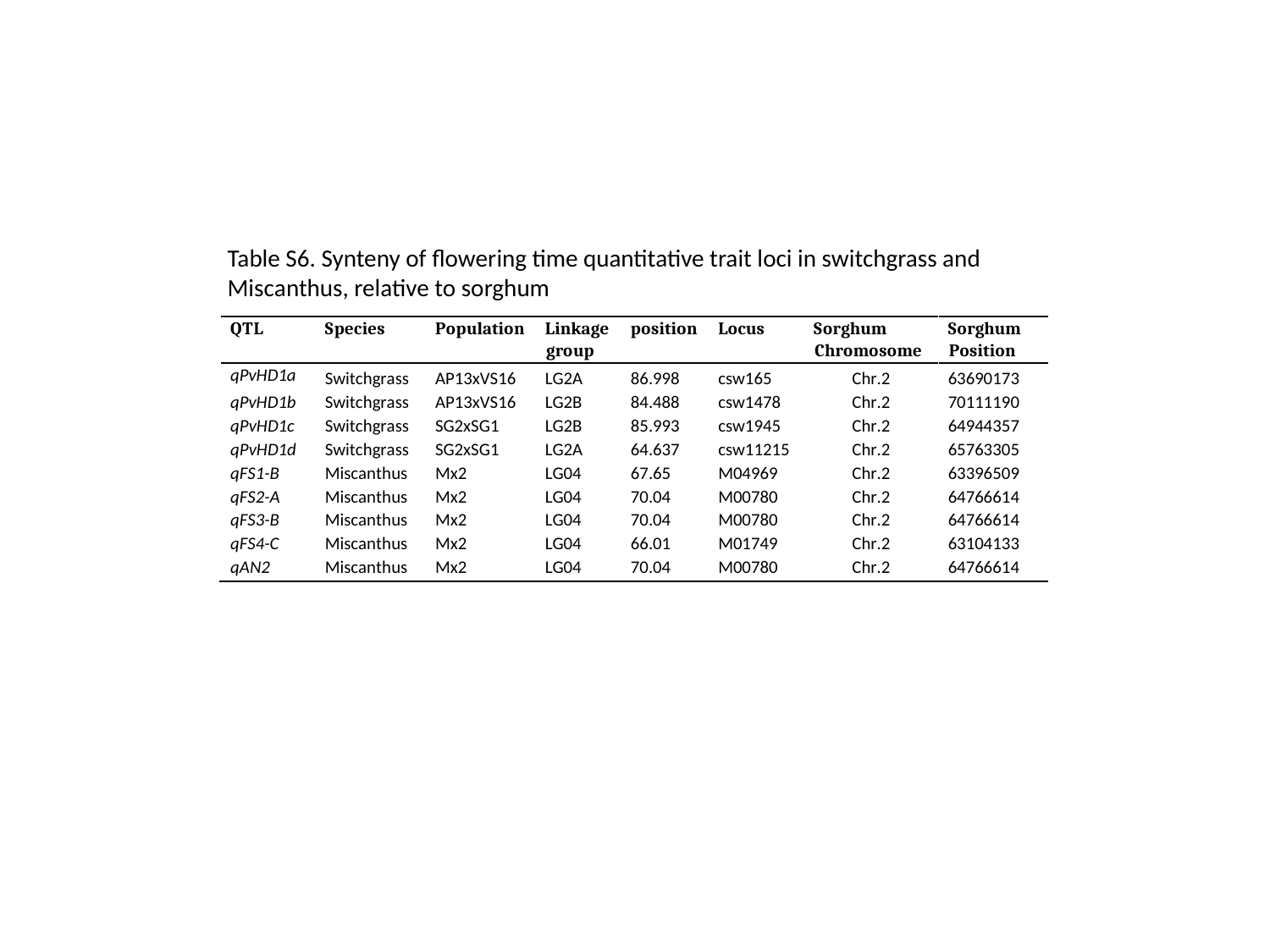

Table S6. Synteny of flowering time quantitative trait loci in switchgrass and Miscanthus, relative to sorghum
